# SNX14 deficiency-induced defective axonal mitochondrial transport in Purkinje cells underlies cerebellar ataxia and can be reversed by valproate

**DOI:** 10.1093/nsr/nwab024

**Published:** 2021-02-10

**Authors:** Hongfeng Zhang, Yujuan Hong, Weijie Yang, Ruimin Wang, Ting Yao, Jian Wang, Ke Liu, Huilong Yuan, Chaoqun Xu, Yuanyuan Zhou, Guanxian Li, Lishan Zhang, Hong Luo, Xian Zhang, Dan Du, Hao Sun, Qiuyang Zheng, Yun-Wu Zhang, Yingjun Zhao, Ying Zhou, Huaxi Xu, Xin Wang

**Affiliations:** State Key Laboratory of Cellular Stress Biology, Fujian Provincial Key Laboratory of Neurodegenerative Disease and Aging Research, Institute of Neuroscience, School of Medicine, Xiamen University, Xiamen 361102, China; State Key Laboratory of Cellular Stress Biology, Fujian Provincial Key Laboratory of Neurodegenerative Disease and Aging Research, Institute of Neuroscience, School of Medicine, Xiamen University, Xiamen 361102, China; State Key Laboratory of Cellular Stress Biology, Fujian Provincial Key Laboratory of Neurodegenerative Disease and Aging Research, Institute of Neuroscience, School of Medicine, Xiamen University, Xiamen 361102, China; State Key Laboratory of Cellular Stress Biology, Fujian Provincial Key Laboratory of Neurodegenerative Disease and Aging Research, Institute of Neuroscience, School of Medicine, Xiamen University, Xiamen 361102, China; State Key Laboratory of Cellular Stress Biology, Fujian Provincial Key Laboratory of Neurodegenerative Disease and Aging Research, Institute of Neuroscience, School of Medicine, Xiamen University, Xiamen 361102, China; State Key Laboratory of Cellular Stress Biology, Fujian Provincial Key Laboratory of Neurodegenerative Disease and Aging Research, Institute of Neuroscience, School of Medicine, Xiamen University, Xiamen 361102, China; National Institute for Data Science in Health and Medicine, School of Medicine, Xiamen University, Xiamen 361102, China; State Key Laboratory of Cellular Stress Biology, Fujian Provincial Key Laboratory of Neurodegenerative Disease and Aging Research, Institute of Neuroscience, School of Medicine, Xiamen University, Xiamen 361102, China; National Institute for Data Science in Health and Medicine, School of Medicine, Xiamen University, Xiamen 361102, China; State Key Laboratory of Cellular Stress Biology, Fujian Provincial Key Laboratory of Neurodegenerative Disease and Aging Research, Institute of Neuroscience, School of Medicine, Xiamen University, Xiamen 361102, China; State Key Laboratory of Cellular Stress Biology, Fujian Provincial Key Laboratory of Neurodegenerative Disease and Aging Research, Institute of Neuroscience, School of Medicine, Xiamen University, Xiamen 361102, China; State Key Laboratory of Cellular Stress Biology, Fujian Provincial Key Laboratory of Neurodegenerative Disease and Aging Research, Institute of Neuroscience, School of Medicine, Xiamen University, Xiamen 361102, China; State Key Laboratory of Cellular Stress Biology, Fujian Provincial Key Laboratory of Neurodegenerative Disease and Aging Research, Institute of Neuroscience, School of Medicine, Xiamen University, Xiamen 361102, China; State Key Laboratory of Cellular Stress Biology, Fujian Provincial Key Laboratory of Neurodegenerative Disease and Aging Research, Institute of Neuroscience, School of Medicine, Xiamen University, Xiamen 361102, China; Cancer Research Center, Department of Stomatology, School of Medicine, Xiamen University, Xiamen 361102, China; State Key Laboratory of Cellular Stress Biology, Fujian Provincial Key Laboratory of Neurodegenerative Disease and Aging Research, Institute of Neuroscience, School of Medicine, Xiamen University, Xiamen 361102, China; State Key Laboratory of Cellular Stress Biology, Fujian Provincial Key Laboratory of Neurodegenerative Disease and Aging Research, Institute of Neuroscience, School of Medicine, Xiamen University, Xiamen 361102, China; State Key Laboratory of Cellular Stress Biology, Fujian Provincial Key Laboratory of Neurodegenerative Disease and Aging Research, Institute of Neuroscience, School of Medicine, Xiamen University, Xiamen 361102, China; State Key Laboratory of Cellular Stress Biology, Fujian Provincial Key Laboratory of Neurodegenerative Disease and Aging Research, Institute of Neuroscience, School of Medicine, Xiamen University, Xiamen 361102, China; National Institute for Data Science in Health and Medicine, School of Medicine, Xiamen University, Xiamen 361102, China; State Key Laboratory of Cellular Stress Biology, Fujian Provincial Key Laboratory of Neurodegenerative Disease and Aging Research, Institute of Neuroscience, School of Medicine, Xiamen University, Xiamen 361102, China; State Key Laboratory of Cellular Stress Biology, Fujian Provincial Key Laboratory of Neurodegenerative Disease and Aging Research, Institute of Neuroscience, School of Medicine, Xiamen University, Xiamen 361102, China

**Keywords:** sorting nexin 14, cerebellar ataxia, Purkinje cell degeneration, mitochondrial dysfunction, axonal transport, valproate

## Abstract

Loss-of-function mutations in sorting nexin 14 (*SNX14*) cause autosomal recessive spinocerebellar ataxia 20, which is a form of early-onset cerebellar ataxia that lacks molecular mechanisms and mouse models. We generated *Snx14*-deficient mouse models and observed severe motor deficits and cell-autonomous Purkinje cell degeneration. SNX14 deficiency disrupted microtubule organization and mitochondrial transport in axons by destabilizing the microtubule-severing enzyme spastin, which is implicated in dominant hereditary spastic paraplegia with cerebellar ataxia, and compromised axonal integrity and mitochondrial function. Axonal transport disruption and mitochondrial dysfunction further led to degeneration of high-energy-demanding Purkinje cells, which resulted in the pathogenesis of cerebellar ataxia. The antiepileptic drug valproate ameliorated motor deficits and cerebellar degeneration in *Snx14*-deficient mice via the restoration of mitochondrial transport and function in Purkinje cells. Our study revealed an unprecedented role for SNX14-dependent axonal transport in cerebellar ataxia, demonstrated the convergence of SNX14 and spastin in mitochondrial dysfunction, and suggested valproate as a potential therapeutic agent.

## INTRODUCTION

Ataxia is a debilitating nervous system disorder that manifests as impaired autonomous coordination of limb movements. Etiologically, ataxia is a complex disease that is induced by a combination of environmental and genetic factors [1]. Cumulative evidence indicates that genetic factors play key roles in ataxia [2]. Hereditary ataxia is classified into autosomal dominant, autosomal recessive and X-linked forms. The incidence of autosomal recessive cerebellar ataxia (ARCA) is approximately three cases per 100 000 individuals, with overlapping symptoms, such as cerebellar atrophy or hypoplasia [3]. Cerebellar Purkinje cell dysfunction or degeneration is a primary pathological event in patients and animal models with cerebellar ataxia [4–6]. Clinically, patients with ARCA exhibit gait irregularities, imbalance, difficulties with swallowing, dyskinesia and other features related to motor function. Despite the severity of impairment associated with ARCA, its mechanisms remain elusive, and no specific treatments have been described.

Recent studies [7,8] reported the identification of a new type of early-onset ARCA, autosomal recessive spinocerebellar ataxia 20 (SCAR20). SCAR20 patients exhibit early-onset cerebellar atrophy, severe motor ataxia, moderate to severe mental retardation and autistic behavior. Whole-exome sequencing analysis identified the sorting nexin 14 (*SNX14*) gene as a candidate gene locus for SCAR20 onset, and *SNX14* loss-of-function mutations account for approximately 9.9% of early-onset cerebellar atrophy and ataxia cases, which represents a higher percentage than any other potential genes related to cerebellar atrophy, such as *GRID2* (2.47%), *NPC1* (1.23%) and *SETX* (1.23%) [7]. SNX14 is a member of the sorting nexin (SNX) family, which consists of 33 related proteins comprised of a conserved phosphoinositol-binding phox homology (PX) domain that facilitates the regulation of vesicular transport and protein sorting [9]. Our work and other studies demonstrated that SNXs play important roles in multiple neurodegenerative diseases [10–14]. Recent studies also indicated that SNX14 regulated lipid metabolism in fibroblasts from individuals with *SNX14* mutations and in *SNX14*-depleted cell lines [15]. Although SNX14 is highly expressed in the central nervous system [16–18], the pathophysiological functions of SNX14 in SCAR20 onset are largely unknown, and therapeutic strategies for SCAR20 have not been elucidated.

The present study showed that *Snx14*-deficient mouse models recapitulated the pathological manifestations of SCAR20 patients, in which *Nestin-Cre*-mediated SNX14 deletion in neurons and glia induced defects in axonal transport, mitochondrial dysfunction and progressive loss of Purkinje cells. Purkinje cell degeneration aggravated cerebellar degeneration via enhanced microglial activation and inflammatory cytokine production. Notably, valproate (VPA) treatment in *Snx14*-deficient mice reversed impairments in motor coordination, Purkinje cell death and mitochondrial dysfunction. Together, we established mouse models that reproduced SCAR20 pathology, described the associated mechanisms underlying SCAR20 pathogenesis, and identified the use of the antiepileptic agent VPA as a potential corrective therapy for cerebellar atrophy and ataxia.

## RESULTS

### SNX14 deficiency causes progressive cerebellar ataxia and atrophy

*Snx14^flox/flox^* (*Snx14^f/f^*) mice were generated via the targeted deletion of exons 9–12 of the murine *Snx14* gene, where most homologous human mutations in SCAR20 were previously identified (Fig. S1A). Constitutive *Snx14* deletion produced no viable *Snx14^–/–^* offspring in multiple crosses between heterozygous breeding pairs (*Snx14^+/–^*× *Snx14^+/–^*) (Fig. S1B). Embryonic lethality was confirmed in pups prior to birth at embryonic day 12.5 (E12.5) (Fig. S1C). Therefore, we generated a conditional *Snx14*-knockout mouse model in neurons and glia by crossing *Snx14^f/f^* mice with a *Nestin-Cre* line. *Snx14^f/f^; Nestin-Cre* (cKO) mice showed normal viability and fertility, and we confirmed that SNX14 expression was ablated in the central nervous system and retained in other tissues (Fig. S1D–F).

**Figure 1.**
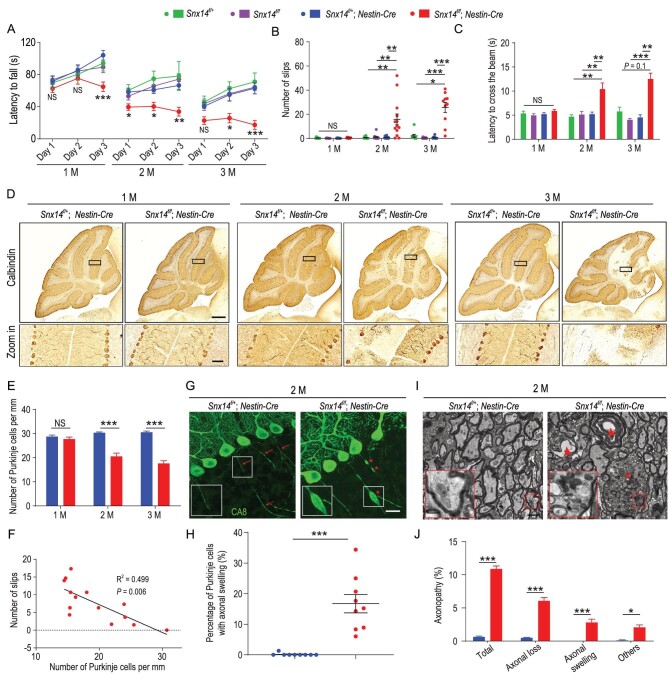
Progressive motor coordination deficits and Purkinje cell degeneration in *Snx14*-deficient mice. (A–C) Motor coordination in *Snx14^f/+^, Snx14^f/f^, Snx14^f/+^; Nestin-Cre* and *Snx14^f/f^; Nestin-Cre* mice as determined by the rotarod and balance beam tests. (A) Quantification of the latency to fall in the rotarod test. *n *= 8–16 mice per genotype per age. (B) The number of foot slips and (C) the latency to traverse the beam in balance beam tests. *n* = 8–13 per genotype per age. (D) Immunohistochemical (IHC) labeling of calbindin^+^ Purkinje cells in *Snx14^f/+^; Nestin-Cre* and *Snx14^f/f^; Nestin-Cre* mouse cerebella. Scale bars = 500 μm (top) and 50 μm (bottom). (E) Quantification of cerebellar Purkinje cells. *n* = 3 mice (total 8–9 slices). (F) The correlation between Purkinje cell density and the number of foot slips in balance beam tests. *n* = 14 *Snx14^f/f^; Nestin-Cre* mice. (G) Immunofluorescence staining to detect CA8-positive Purkinje cells in the mouse cerebellum. Intact axons (arrows) and swollen axons (asterisks) are indicated. Scale bar = 20 μm. (H) The percentage of Purkinje cells with swollen axons in *Snx14^f/+^; Nestin-Cre* mice (*n* = 3 mice, 1175 cells) and *Snx14^f/f^; Nestin-Cre* mice (*n* = 3 mice, 438 cells). (I) TEM analysis of Purkinje cell axons. The asterisk indicates a swollen axon, and arrowheads indicate axonal loss associated with a collapsed myelin sheath. Scale bar = 2 μm. (J) The percentage of Purkinje cells with axonopathies (axonal swelling, axonal loss and other types). *n *= 3–4 mice. A total of 300–500 axons per mouse were scored. Male animals were used in (A–J). 1 M, 2 M and 3 M represent 1 month, 2 months and 3 months of age, respectively. All data represent means ± SEM. *P* values were determined using repeated-measures ANOVA with Bonferroni's *post hoc* analysis in (A), the Kruskal-Wallis test with Dunn's *post hoc* analysis in (B) and (C), Student's *t* test in (E), (H) and (J), and Spearman's rank correlation in (F). NS, not significant; ^*^P < 0.05; ^*^^*^P < 0.01; ^*^^*^^*^P < 0.001.

*Snx14* cKO mice had a similar lifespan (Fig. S1G) and body weight (Fig. S1H) as *Snx14^f/+^; Nestin-Cre* mice (cHet). To evaluate the effects of *Snx14* deletion in the central nervous system (CNS) on motor coordination, we compared rotarod and balance beam performance in cKO and wild-type (WT) animals. We found that cKO mice showed mild impairment in motor coordination from 1 month of age, which progressively deteriorated in animals at 2 and 3 months according to rotarod tests (Figs. 1A and S1I). Similar deficits in motor coordination were observed in male and female cKO mice in the balance beam tests (Fig. 1B and C, Fig. S1J and K and Movie S1).

We also examined morphological changes in the brain and observed that the cerebella of 3-month-old cKO mice were smaller in size and weight than those of control mice (Fig. S2A). Further histological analyses revealed a reduction in the thickness of the cerebellar molecular layer (ML), which is a region enriched with Purkinje cell dendrites (Fig. S2B), but no obvious differences were observed in the thickness of the granular layer (GL) (Fig. S2C), the thickness of the cerebral cortex or the number of NeuN^+^ neurons in the cerebral cortex (Fig. S2D and E), which suggests that Purkinje cells within the cerebellum are specifically susceptible to SNX14 deletion. These results demonstrate that SNX14 deficiency induces progressive ataxia phenotypes and cerebellar atrophy.

### SNX14 deficiency induces progressive cerebellar Purkinje cell degeneration and microglial activation

Although no changes in Purkinje cell pathology were observed in 1-month-old cKO mice, ∼30% and ∼40% reductions in Purkinje cells were observed in 2- and 3-month-old cKO mice, respectively, compared to cHet mice (Fig. 1D and E). We examined whether *SNX14* haploinsufficiency affected Purkinje cell survival using immunohistochemical analysis and found a normal density of Purkinje cells in cHet mice compared to littermate *Snx14^f/f^* (WT) mice (Fig. S2F). Notably, enhanced neuropathological effects were observed in female cKO mice compared to males (Fig. S2G). No change in cerebellar granule cell number was observed at the age of 3 months, as quantified by NeuN levels (Fig. S2H). We observed that the Purkinje cell number negatively correlated with foot slips in balance beam tests (Fig. 1F), which suggests SNX14-dependent Purkinje cell degeneration in motor impairment.

To further characterize SNX14-dependent morphological alterations in Purkinje cells, we performed immunofluorescence staining using the Purkinje cell marker carbonic anhydrase 8 (CA8) and observed swollen axons in ∼16% CA8-positive Purkinje cells in 2-month-old cKO mice (Fig. 1G and H). We also observed aberrant accumulation of mitochondria, lysosomes and other organelles in swollen axons using transmission electron microscopy (TEM) (Fig. 1I, Fig. S3A and B). In addition to axonal swelling, other axonopathological features (Fig. 1I and J) and hypomyelination (Fig. S3C–E) were observed in cKO mice. Axonal swelling was evident in cKO mice from 1 month of age (Fig. S3F and G), which suggests that axonopathy in Purkinje cells is an early event in SCAR20 pathogenesis and onset.

To determine whether Purkinje cell degeneration induced neuroinflammation, we performed double immunostaining using ionized calcium binding adaptor molecule 1 (IBA1) and CA8 antibodies. We observed significant microglial proliferation and activation associated with Purkinje cell degeneration in the cerebella of 2-month-old but not 1-month-old cKO mice (Fig. S4A–D). Purkinje cell number and microglial abundance were inversely correlated (Fig. S4E). We subsequently observed an upregulation of the mRNA expression of proinflammatory cytokines, such as tumor necrosis factor (*Tnf*) and interleukin 1β (*Ilb*), in *Snx14-*deficient mouse cerebella using quantitative reverse transcription polymerase chain reaction (qRT-PCR) analysis (Fig. S4F).

### SNX14 deficiency induces cell-autonomous death in Purkinje cells

**Figure 2.**
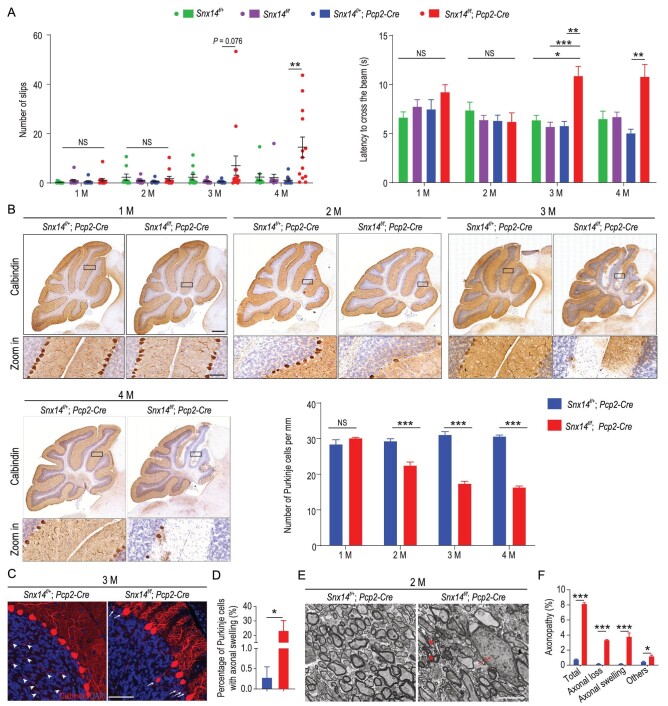
SNX14 deficiency induces cell-autonomous degeneration of Purkinje cells. (A) Quantification of foot slips (left) and the latency to traverse the beam (right) in balance beam tests in male mice (*n* = 8–14 mice). (B) IHC labeling and quantification of calbindin^+^ Purkinje cells in the cerebella of female *Snx14^f/+^; Pcp2-Cre* and *Snx14^f/f^; Pcp2-Cre* mice. Scale bars = 500 μm (top) and 50 μm (bottom), *n* = 3 mice. (C) Immunofluorescence staining of calbindin^+^ Purkinje cells in female *Snx14^f/+^; Pcp2-Cre* and *Snx14^f/f^; Pcp2-Cre* mouse cerebella. Arrowheads (left) indicate intact axons, and arrows (right) indicate swollen axons. Scale bar = 50 μm. (D) The percentage of Purkinje cells with swollen axons in *Snx14^f/+^; Pcp2-Cre* mice (*n* = 3 mice, 399 cells) and *Snx14^f/f^; Pcp2-Cre* mice (*n* = 3 mice, 174 Purkinje cells). (E) Representative TEM images of transverse cerebellar tissue sections. The arrow indicates a swollen axon, and asterisks indicate axonal loss with a collapsed myelin sheath. Scale bar = 5 μm. (F) The percentage of Purkinje cells with axonopathies (axonal loss, axonal swelling and other types). *n* = 3–4 mice per genotype; 300–500 axons per mouse were scored. 1 M, 2 M, 3 M and 4 M indicate 1–4 months of age, respectively. All data represent means ± SEM. *P* values were determined using the Kruskal-Wallis test with Dunn's *post hoc* analysis in (A) and Student's *t* test in (B), (D) and (F). NS, not significant; ^*^P < 0.05; ^*^^*^P < 0.01; ^*^^*^^*^P < 0.001.

*Snx14* mRNA is predominately expressed in the Purkinje cells of the mouse cerebellum (Fig. S5). Therefore, we hypothesized that SNX14 was essential for Purkinje cell survival. To test this hypothesis, we generated a cell-specific *Snx14* KO mouse model in Purkinje cells by crossing *Snx14^f/f^* with a *Pcp2-Cre* transgenic mouse line. We first evaluated motor coordination using balance beam tests. Little or no impairment in motor coordination was observed in 1- and 2-month-old male *Snx14^f/f^; Pcp2-Cre* animals, but the 3- and 4-month-old *Snx14^f/f^; Pcp2-Cre* mice required a longer time to cross the balance beam and exhibited more foot slips than control mice (Fig. 2A). Notably, impaired motor coordination was enhanced in female *Snx14^f/f^; Pcp2-Cre* mice compared to male mice (Fig. S6A and B).

Similar to the cKO mice, male and female *Snx14^f/f^; Pcp2-Cre* mice exhibited a progressive loss of Purkinje cells with age (Fig. 2B and Fig. S6C). More than 20% of Purkinje cells in 3-month-old *Snx14^f/f^; Pcp2-Cre* mice exhibited swollen axons (Fig. 2C and D). Similar to cKO mice, ultrastructural analysis revealed significant axonopathy in *Snx14^f/f^; Pcp2-Cre* mice (Fig. 2E and F) and glial activation in 3- and 4-month-old *Snx14^f/f^; Pcp2-Cre* mice (Fig. S6D–K). These results indicate that cell-autonomous Purkinje cell death and axon degeneration are pathological features of SCAR20.

To determine whether hypomyelination contributed to the neuropathological features observed in cKO mice, we selectively deleted *Snx14* in oligodendrocyte lineage cells by breeding *Snx14^f/f^* mice with *Olig1-Cre* transgenic mice. Although cerebellar hypomyelination was evident in transgenic *Snx14^f/f^; Olig1-Cre* mice (Fig. S7A–C), no alterations in motor coordination (Fig. S7D and E) and only a slight reduction (∼15% loss) in Purkinje cells (Fig. S7F–H) were observed in these animals up to 6 months of age. Taken together, these results indicate that Purkinje cell degeneration likely drives SCAR20 pathogenesis, with little or no contribution from oligodendrocyte dysfunction.

### Mitochondrial dysfunction in *Snx14*-deficient Purkinje cells

**Figure 3.**
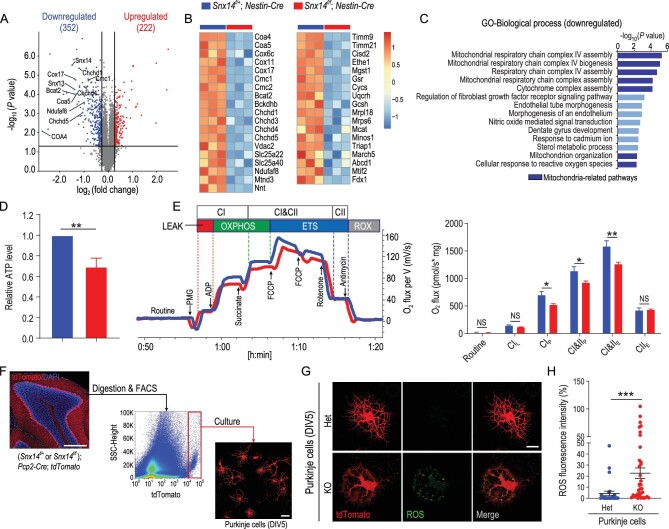
Mitochondrial dysfunction in the *Snx14*-deficient mouse cerebellum. (A) Proteomic analysis of the mouse cerebellum: volcano plot illustrating upregulated (red) and downregulated (blue) proteins in 1-month-old *Snx14^f/f^; Nestin-Cre* compared to *Snx14^f/+^; Nestin-Cre* mouse cerebella (fold change > 1.2, *P* value < 0.05). *n* = 3 samples. Each sample was derived from cerebellar tissue pooled from three mice of the same genotype. (B) Heatmap depicting downregulated mitochondrial proteins in the *Snx14^f/f^; Nestin-Cre* mouse cerebellum. (C) GO analysis of downregulated proteins in the *Snx14^f/f^; Nestin-Cre* mouse cerebellum. Mitochondria-related pathways are marked in dark blue. (D) Quantification of ATP levels in 1-month-old *Snx14^f/+^; Nestin-Cre* and *Snx14^f/f^; Nestin-Cre* mouse cerebella. (E) Representative respirometric traces from cerebellar homogenates in 1.5-month-old *Snx14^f/+^; Nestin-Cre* (*n* = 10 mice) and *Snx14^f/f^; Nestin-Cre* mice (*n* = 15 mice) using high-resolution FluoRespirometry. (F) Experimental outline, FACS isolation and *in vitro* culture of tdTomato^+^ Purkinje cells from P6 *Snx14^f/+^; Pcp2-Cre; tdTomato* and *Snx14^f/f^; Pcp2-Cre; tdTomato* cerebella. Scale bars = 500 μm (left) and 50 μm (right). (G) Visualization of ROS production in *Snx14* Het and KO Purkinje cells (DIV5) using DCFH-DA (green). Scale bar = 20 μm. (H) Quantification of ROS fluorescence intensity in Het (*n* = 28 cells) and KO (*n* = 38 cells) Purkinje cells. Male animals were used in (A–E). All data represent means ± SEM. *P* values were determined using the Student's *t* test in (D), (E) and (H). NS, not significant; ^*^P < 0.05; ^*^^*^P < 0.01; ^*^^*^^*^P < 0.001.

To determine the underlying molecular mechanism of Purkinje cell degeneration in SCAR20, we characterized global proteomic expression profiles in 1-month-old (the early phase of disease progression) cHet and cKO mouse cerebella using tandem mass tag (TMT)-based quantitative proteomics. A total of 222 significantly upregulated and 352 downregulated differentially expressed proteins (DEPs) were identified (Fig. 3A). We confirmed the expression of several downregulated proteins from the proteomic study using immunoblot analysis, such as CHCHD4 (mitochondria-related), SNX1 (protein sorting-related), MBP and MOG (myelin-related). As expected, the expression levels of these proteins were markedly reduced in the cerebellum of cKO mice compared to cHet mice (Fig. S8).

Notably, we observed an enrichment of mitochondrial components in downregulated DEPs (Fig. 3B). Gene ontology (GO) analysis of relevant biological process (BP) pathways revealed a downregulation of proteins related to mitochondrial respiratory chain complex biogenesis/assembly and the cellular response to reactive oxygen species (ROS) (Fig. 3C). These results suggest a role for SNX14 in homeostatic mitochondrial function. Therefore, we examined whether SNX14 deficiency altered mitochondrial function in the mouse cerebellum. As expected, adenosine triphosphate (ATP) production was markedly reduced in the cerebellum of cKO mice (Fig. 3D). We also observed a marked reduction in CI- and CII-linked oxidative phosphorylation (OXPHOS) (CI_P_ and CI&II_P_) using high-resolution FluoRespirometry and a reduced maximal capacity of the mitochondrial electron transport system (ETS) (CI&II_E_) in cKO compared to cHet mouse cerebella (Fig. 3E). These results indicate that *Snx14* deficiency impairs mitochondrial respiration in the cerebellum.

Dysfunction of the mitochondrial respiratory chain generally leads to the overproduction of mitochondrial ROS, which induces cellular damage [19]. To determine whether SNX14 deletion affected ROS production in Purkinje cells, we crossed *Snx14^f/f^; Pcp2-Cre* mice with a red fluorescent protein reporter (R26-LSL-tdTomato) mouse line to obtain *Snx14^f/f^; Pcp2-Cre; tdTomato* (KO) and *Snx14^f/+^; Pcp2-Cre; tdTomato* (Het) mice to induce exclusive tdTomato expression in cerebellar Purkinje cells (Fig. S9A). The tdTomato-expressing Purkinje cells in newborn mice (P6) were isolated using flow cytometry sorting (FACS) and cultured *in vitro* for 5 days (Fig. 3F). We used the fluorescent probe 2^′^,7^′^-dichlorofluorescin-diacetate (DCFH-DA) to quantify ROS production and observed increased ROS production in Snx14 KO cells compared to Snx14 Het Purkinje cells (Fig. 3G and H). Snx14 KO Purkinje cells exhibited reduced cellular sizes (Fig. S9B) and branch complexity (Fig. S9C and D). Together, these results indicate that Snx14 deficiency impairs cerebellar mitochondrial function in Purkinje cells.

### SNX14 deficiency impairs mitochondrial transport in axons and destabilizes spastin

**Figure 4.**
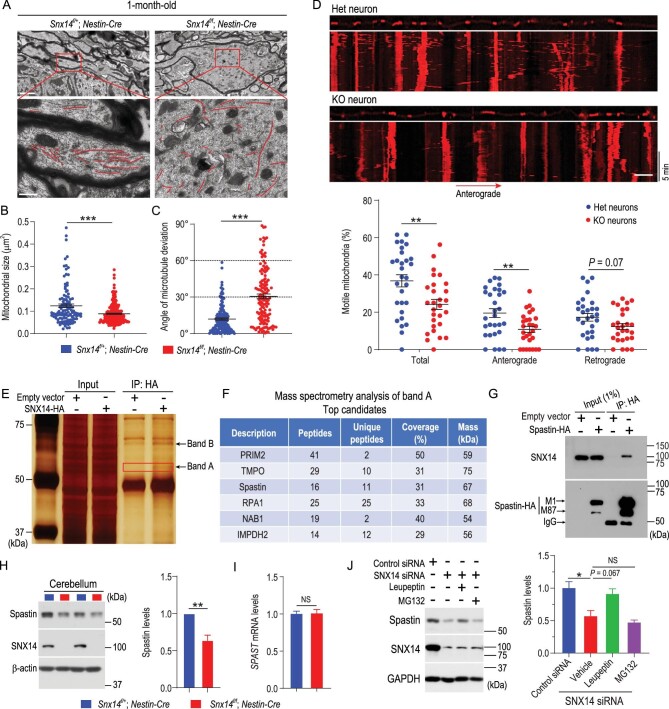
SNX14 deficiency disrupts microtubule organization and mitochondrial transport in axons. (A) TEM analysis of longitudinal sections from Purkinje cell axons in male *Snx14^f/+^; Nestin-Cre* and *Snx14^f/f^; Nestin-Cre* mice. Microtubules are traced with red lines (bottom). Scale bars = 2 μm (top) and 0.5 μm (bottom). (B) Quantification of mitochondrial size and (C) orientation (angle of microtubule deviation) in swollen axons from Purkinje cells. Each plot represents a single microtubule filament or mitochondrion (at least nine TEM images per genotype were analyzed and pooled for statistical analysis). *n* = 3–5 mice per genotype. (D) Representative kymographs and quantitative analysis of mitochondrial transport in axons from *Snx14^f/+^; Nestin-Cre* (Het) and *Snx14^f/f^; Nestin-Cre* (KO) neurons. Scale bar = 5 μm. *n* = 28–29 cells in each group. (E) Two specific bands (bands A and B, indicated by arrows) were identified in HA-immunoprecipitates from HEK293T cell lysates transfected with SNX14-HA (silver stain). (F) Six SNX14-interacting proteins were identified using mass spectrometry analysis from the excised band A in (E) (red box). (G) Co-IP between exogenously expressed spastin-HA (isoforms M1 and M87) and endogenous SNX14 protein. IP, immunoprecipitation. (H) Immunoblot analysis of spastin in cerebella from 1-month-old mice. Signal intensities from immunoblots were normalized to β-actin. *n* = 5. (I) *SPAST* mRNA levels quantified using qRT-PCR in the cerebellum of 1-month-old mice. *n* = 6 mice. (J) Immunoblot analysis of spastin in cell lysates from SNX14-depleted HEK293T cells treated with a lysosomal inhibitor (leupeptin) or proteasomal inhibitor (MG132). n = 3. All data represent means ± SEM. *P* values were determined using Student's *t* test in (B), (C), (D), (H) and (I), and one-way ANOVA with Tukey's *post hoc* analysis in (J). NS, not significant; ^*^P < 0.05; ^*^^*^P < 0.01; ^*^^*^^*^P < 0.001.

We observed an accumulation of smaller mitochondria (Fig. 4A and B) and marked microtubule disorganization (Fig. 4A and C) in the swollen axons from *Snx14*-deficient Purkinje cells. Because axonal cargo transport is dependent on linear microtubule organization, we hypothesized that SNX14 deficiency would disrupt the microtubule-based transport of organelles, such as mitochondria. Microtubule misalignment and disorganization ultimately induce altered mitochondrial distribution along the axon and cause mitochondrial dysfunction. Therefore, we monitored mitochondrial motility in the axons of *Snx14*-deficient neurons using real-time microscopic imaging. As expected, markedly fewer motile mitochondria were observed in the axons of *Snx14* KO neurons than in Het neurons (Fig. 4D and Movie S2). Anterograde and retrograde mitochondrial transport was compromised. However, SNX14 deficiency appeared to affect anterograde transport to a greater extent (Fig. 4D).

As a member of the SNX family, SNX14 likely functions as an intracellular trafficking regulator via interactions with other proteins. To identify SNX14-binding proteins, HEK293T cells were transfected with a hemagglutinin (HA)-tagged SNX14 (SNX14-HA) plasmid and then subjected to immunoprecipitation (IP) using an anti-HA antibody. We identified multiple potential SNX14-interacting proteins using proteomic analysis (Fig. 4E and F and Table S2), including the microtubule-severing protein spastin, which is required to maintain axonal integrity [20]. Similar to *SNX14, SPAST* (the spastin coding gene) haploinsufficiency also induces axonal swelling and impairs mitochondrial transport [21,22]. We confirmed the interactions between spastin and SNX14 using co-IP (Fig. 4G) and *in vitro* pulldown assays (Fig. S10A) and demonstrated that spastin and SNX14 formed a complex. Moreover, we observed colocalization of SNX14 and spastin and that SNX14-spastin puncta partially colocalized with the lipid droplet marker BODIPY in HeLa cells (Fig. S10B). To determine the critical interaction domains of each protein, we performed domain mapping assays using truncated spastin and SNX14 protein fragments. We found that deletion of the N-terminal domain (1–86 aa, M87 form) of spastin completely abolished the interaction between spastin and SNX14, and deletion of the hydrophobic region (HR) (49–80 aa, ΔHR) largely disrupted the interaction (Fig. S10B). However, the microtubule interacting and endosomal trafficking (MIT) domain and the microtubule-binding (MBD) and AAA ATPase (AAA) domains of spastin were not essential for the spastin-SNX14 interaction (Fig. S10C). Moreover, we found that the PX-associated domain A (PXA), regulator of G protein signaling (RGS), PX and PX-associated domain C (PXC) domains of the SNX14 protein were not required for the SNX14-spastin interaction (Fig. S10D); however, an SNX14 N-terminal fragment with transmembrane domains (SNX14-TM, aa 1–130) can interact with M1 spastin (Fig. S10E). Together, these results indicated that the N-terminal fragment of M1 spastin (aa1–86) and the N-terminal fragment of SNX14 (aa1–130) are required for their interaction.

Spastin protein levels were downregulated in the cerebella of cKO mice compared to cHet mice (Fig. 4H), but *SPAST* mRNA levels were not changed (Fig. 4I), which suggests that SNX14 regulates spastin expression posttranscriptionally. We observed that enhanced spastin turnover with SNX14 deficiency was largely reversed by the inhibition of lysosomal, rather than proteasomal, protein degradation (Fig. 4J). To determine whether the restoration of spastin protein expression rescued defective mitochondrial transport in the axons of *Snx14* KO neurons, we infected *Snx14* KO neurons with recombinant SPAST adeno-associated virus (rAAV) and observed that M1 spastin overexpression largely restored mitochondrial motility in *Snx14* KO neurons (Fig. S11). These results indicate that SNX14 deficiency-induced spastin degradation accounts for defective mitochondrial transport in axons.

### VPA treatment ameliorates the pathological effects associated with SNX14 deficiency

**Figure 5.**
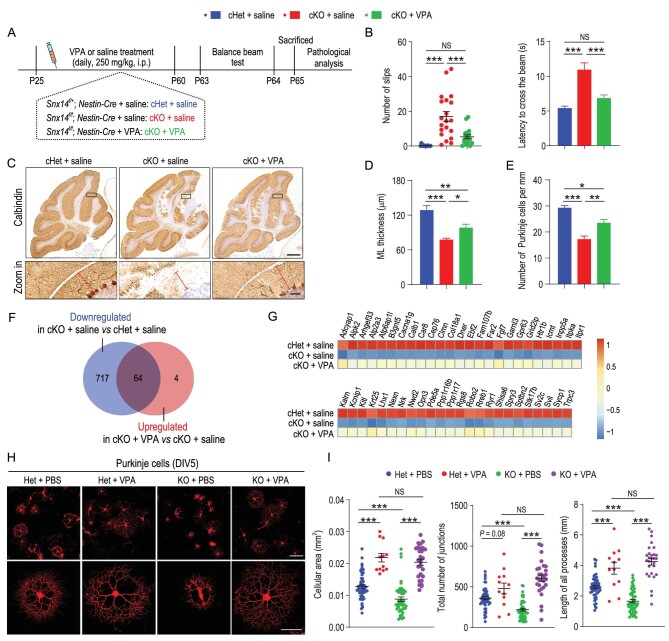
VPA treatment ameliorates cerebellar ataxia and Purkinje cell death in *Snx14*-deficient mice. (A) Schematic timeline of VPA rescue experiments. The three treatment groups in the study include: *Snx14^f/+^; Nestin-Cre* + saline (cHet + saline), *Snx14^f/f^; Nestin-Cre* + saline (cKO + saline) and *Snx14^f/f^; Nestin-Cre *+ VPA (cKO + VPA). Mice (P25) were intraperitoneally (i.p.) injected with saline or VPA (250 mg/kg/day) for 5 weeks, followed by behavioral and pathological analyses. (B) Quantification of mouse foot slips (left) and the latency to traverse the beam (right) in balance beam tests. *n *= 15–20 mice. (C) Representative images of calbindin-expressing Purkinje cells in cHet + saline, cKO + saline and cKO + VPA mice. Scale bars = 500 μm (top) and 50 μm (bottom). (D) Measurement of cerebellar ML thickness upon VPA treatment. *n* = 3–4 mice. (E) Histological analyses to determine the effects of VPA on Purkinje cell survival. *n *= 3–5 mice. (F and G) Transcriptomic analysis of 2-month-old male cHet + saline, cKO + saline and cKO + VPA mice (fold change > 1.2; adjusted *P* value < 0.1). *n* = 7 mice per group. (F) Venn diagram depicting downregulated genes in the cKO + saline group compared to the cHet + saline group (blue circle) and upregulated genes in the cKO + VPA group relative to the cKO + saline group (red circle); 64 genes in the overlap region indicate downregulated genes with SNX14 deletion restored by VPA treatment. (G) Heatmap depicting Purkinje cell-specific genes within the 64 genes showing VPA-dependent normalization. (H) Representative images of Het and KO Purkinje cells upon saline or VPA treatment. Scale bars = 100 μm (top) and 50 μm (bottom). (I) Quantification of the cellular area (left), total number of junctions (center) and the summed length of all processes (right) in Purkinje cells in the Het + PBS (*n* = 56 cells), Het + VPA (*n* = 12 cells), cKO + PBS (*n* = 46 cells) and cKO + VPA (*n* = 28 cells) groups. Male animals were used in (A–I). All data represent means ± SEM. *P* values were determined using one-way ANOVA with Tukey's *post hoc* analysis. NS, not significant; ^*^P < 0.05; ^*^^*^P < 0.01; ^*^^*^^*^P < 0.001.

VPA is an antiepileptic drug that is also used to treat bipolar disorder. VPA enhanced mitochondrial function in SH-SY5Y cells [23] and axonal remodeling in cultured neurons [24]. Therefore, we determined whether VPA reversed mitochondrial dysfunction and Purkinje cell degeneration in *Snx14* KO mice. Postnatal day 25 (P25) cKO mice were given a daily intraperitoneal dose of VPA (250 mg/kg) or saline for 35 consecutive days and subsequently assayed for behavior and cerebellar physiology (Fig. 5A). The administration of VPA at subchronic levels did not produce any apparent toxic effects, such as weight gain, which was similar between the cKO + VPA and cKO + saline groups (Fig. S12A). Notably, subchronic injections of VPA markedly improved motor coordination in male and female cKO mice in the balance beam tests (Fig. 5B, Fig. S12B and Movie S3). In the cKO mouse cerebellum, VPA treatment restored the thickness of the ML (Fig. 5C and D) and reduced the loss of Purkinje cells (Fig. 5C and E and Fig. S12C). VPA treatment similarly reversed Purkinje cell degeneration in *Snx14^f/f^; Pcp2-Cre* mice (Fig. S12D–F). Transcriptomic analysis revealed that 781 genes were downregulated in the cerebellum of cKO mice compared to cHet animals, and 64 of these genes were partially restored with VPA treatment (Fig. 5F). Notably, most of the restored gene signatures were Purkinje cell-specific (49 of 64) (Fig. 5G and Fig. S12G). The expression profiles of the cKO cerebellum showed robust changes in neuroinflammatory signatures (Fig. S12H), and VPA administration normalized the expression of microglia- and astrocyte-specific genes (Fig. S12I and J). Consistent with the transcriptomic analysis, VPA treatment attenuated microglial proliferation and activation in the cKO cerebellum (Fig. S12K–M). To determine whether VPA specifically conferred neuroprotective effects to Purkinje cells, we treated Purkinje cells with VPA *in vitro* and evaluated the consequent effects on morphology. We found that VPA treatment restored cellular size and branch complexity in *Snx14* KO Purkinje cells (Fig. 5H and I).

Although the pharmacological effects of VPA as an antiepileptic agent are not clear, VPA has broad inhibitory activity toward class I and II histone deacetylases (HDACs) [25]. HDAC inhibitors reversed cytotoxicity in cellular and fly models of Parkinson's disease [26]. Therefore, VPA may confer neuroprotective effects via HDAC inhibition. To test this hypothesis, we evaluated the effects of two widely used HDAC inhibitors, SAHA and panobinostat, on the restoration of SNX14 deficiency-induced motor impairment and found that neither compound ameliorated the motor coordination deficits and Purkinje cell loss in cKO mice (Fig. S13A–F). These results suggest that VPA exerts its rescuing effects in an HDAC-independent manner.

A possible link between SNX14 and lipid metabolism was proposed [15,27–30]. Lipid metabolism malfunction is a major feature of Niemann Pick Disease Type C (NPC) [31], and patients with NPC also exhibit progressive cerebellar degeneration that is similar to SCAR20 [32,33]. Therefore, we examined whether the therapeutic strategy for NPC was beneficial for SCAR20. However, the widely used cholesterol chelator HP-β-CD, which was effective in cell and mouse models of NPC [34,35], failed to ameliorate cerebellar degeneration and motor deficits in *Snx14*-deficient mice (Fig. S13G–I), which suggests that *SNX14* deletion induces SCAR20 by signaling pathways other than regulation of the accumulation of unesterified cholesterol.

### VPA promotes Purkinje cell survival via restoration of mitochondrial function

**Figure 6.**
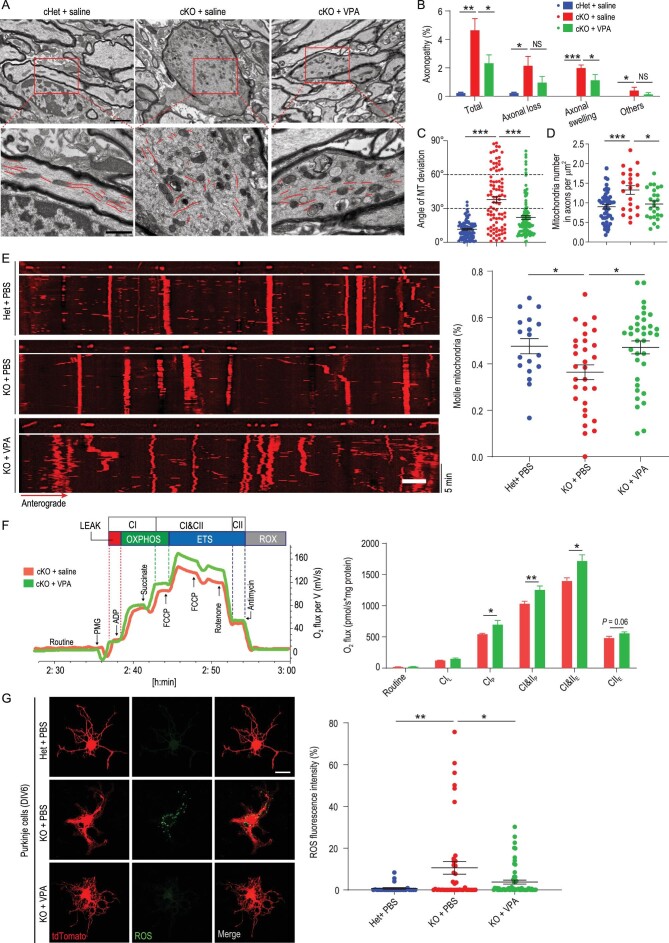
VPA promotes Purkinje cell survival by restoring mitochondrial function. (A) Representative TEM images of longitudinal sections of Purkinje cell axons from cHet + saline, cKO + saline and cKO + VPA mice. Microtubules are traced with red lines (bottom). Scale bars = 2 μm (top) and 1 μm (bottom). (B) Quantification of axonopathies (axonal loss, axonal swelling and other types) of Purkinje cells. *n *= 3–4 mice. A total of 300–500 axons per mouse were scored. (C) Quantification of orientation (angle of microtubule deviation) in swollen axons from Purkinje cells. Each plot represents a single microtubule filament (at least 10 TEM images per genotype were analyzed and pooled for statistical analyses). *n* = 3–5 mice per genotype. (D) Quantification of mitochondria in Purkinje cell axons. *n *= 4 mice. A total of 20–50 axons per group were scored. (E) Representative kymographs and quantification of motile mitochondria in the axons of Het + PBS (*n* = 18 cells), KO + PBS (*n* = 30 cells) and KO + VPA (*n* = 35 cells) neurons. Scale bar = 5 μm. (F) Characterization of mitochondrial function in cKO + saline (*n *= 11 mice) and cKO + VPA (*n* = 11 mice) mouse cerebella using high-resolution FluoRespirometry. (G) Visualization and quantification of ROS production in Het + saline (*n* = 26 cells), KO + saline (*n* = 42 cells) and KO + VPA (*n* = 59 cells) Purkinje cells using DCFH-DA (green). Scale bar = 25 μm. All data represent means ± SEM. *P* values were determined using one-way ANOVA with Tukey's *post hoc* analysis in (B), (C), (D), (E) and (G), and Student's *t* test in (F). NS, not significant; ^*^P < 0.05; ^*^^*^P < 0.01; ^*^^*^^*^P < 0.001.

To examine the mechanisms underlying the therapeutic effects of VPA treatment, we characterized neuronal physiology in *Snx14* KO animals treated with VPA. VPA administration markedly mitigated axonopathies and microtubule disorganization (Fig. 6A–C). VPA treatment also relieved the abnormal axonal accumulation of mitochondria in *Snx14*-deficient Purkinje cells (Fig. 6D). At the molecular level, VPA treatment restored the protein levels of spastin (Fig. S14A) and polymerized α-tubulin (Fig. S14B) in *Snx14* KO neurons. Axonal transport deficits may lead to mitochondrial accumulation. Therefore, we characterized axonal mitochondrial motility using time-lapse microscopy. We found that VPA treatment markedly restored mitochondrial motility in *Snx14* KO neuronal axons (Fig. 6E). We investigated the effects of VPA on cerebellar mitochondrial function and observed significant increases in cerebellar mitochondrial respiratory capacity with VPA treatment, including the respiration levels of CI- and CII-linked OXPHOS and the maximal capacity of the mitochondrial ETS (Fig. 6F). We also observed that VPA treatment decreased ROS production in KO Purkinje cells compared to saline controls (Fig. 6G). Taken together, these results suggest that VPA treatment promotes Purkinje cell survival by reversing mitochondrial dysfunction in *Snx14*-deficient mice.

## DISCUSSION

*SNX14* loss-of-function mutations were identified in patients with cerebellar atrophy, intellectual disabilities and autism [7,8], which suggests a critical role of *SNX14* in brain function. Deletion of *Snx14* in neurons and glia recapitulated human SCAR20 neurological phenotypes, including the impaired coordination of limb movements and Purkinje cell loss previously reported in SCAR20 patients. These results demonstrated that SNX14 dysfunction was sufficient to drive pathological phenotypes with full penetrance and provide a robust model system to characterize SCAR20-associated degeneration.

Multiple cerebellar cell types contribute to the pathogenesis of cerebellar ataxia [36,37]. To identify the most vulnerable cell types in SCAR20, we generated Purkinje cell-specific (*Snx14^f/f^; Pcp2-Cre*) and oligodendrocyte lineage cell-specific (*Snx14^f/f^; Olig1-Cre*) *Snx14* KO mice. Notably, *Snx14^f/f^; Pcp2-Cre* mice recapitulated most of the behavioral and morphological phenotypes of cKO mice. However, *Snx14^f/f^; Olig1-Cre* mice exhibited only hypomyelination without behavioral deficits. Therefore, Purkinje cell degeneration triggers SCAR20 pathogenesis in a cell-autonomous manner. Contributions from other cell types cannot be completely discounted because phenotypes in *Snx14^f/f^; Pcp2-Cre* mice were slightly delayed by ∼2 weeks compared to *Snx14^f/f^; Nestin-Cre* mice. Therefore, the contributions of other cell types in cerebellar ataxia require further investigation.

SNX family proteins play essential roles in intracellular cargo transport, and these proteins are linked to a number of neurodegenerative diseases [38]. Our study revealed that SNX14 was the first SNX protein to regulate mitochondrial transport and function via the modulation of microtubule organization. We found interactions between SNX14 and the microtubule-severing enzyme spastin; SNX14 deficiency disrupted microtubule organization, and reduced spastin expression. Moreover, SPAST haploinsufficiency in human and animal models causes hereditary spastic paraplegia (HSP) with cerebellar ataxia via disrupting microtubule organization and axonal transport of cargoes (including mitochondria) [39,40]. These results demonstrated the convergence of SCAR20 and HSP on a SNX14/spastin pathway (Fig. S15). Future investigations will determine whether SNX14 also regulates its microtubule-severing activity.

Mitochondrial dysfunction is a common pathological feature of neurodegenerative diseases, such as Huntington's disease [41], Parkinson's disease [42], Alzheimer's disease [43], amyotrophic lateral sclerosis [44] and certain types of cerebellar ataxias (SCA1 [45–47] and Friedreich's ataxia [48]). The present study discovered an unidentified convergence of SNX14 and spastin in mitochondrial transport and dysfunction and demonstrated the possibility of the restoration of mitochondrial function by targeting of the pathway in the treatment of cerebellar degeneration.

SNX14 mutations led to increased levels of fatty acids in model animals [28,29] and cholesterol accumulation in human fibroblasts [15]. Moreover, M1 spastin requires its lipid droplet binding N-terminal HR domain to interact with SNX14, suggesting that lipid droplets may provide a niche for the interaction between SNX14 and spastin. SNX14 and spastin could work together at lipid droplets and play a role in fatty acid and lipid metabolism. As mitochondria play a major role in fatty acid oxidation and mitochondrial defects lead to lipid droplet accumulation [49], SNX14 deficiency-mediated mitochondrial dysfunction could also disrupt fatty acid oxidation and lipid droplet accumulation. Our study revealed that HP-β-CD, a potential therapy for NPC [34,35], failed to ameliorate cerebellar degeneration in *Snx14*-deficient mice, which suggests that *SNX14* deletion may lead to Purkinje cell degeneration through a cholesterol-independent mechanism. Future investigations are required to determine whether SNX14 regulates the metabolism of neutral lipids other than cholesterol.

VPA is traditionally used as an antiepileptic agent [50], and it was originally discovered to affect gamma-Aminobutyric acid (GABA) levels, block voltage-gated sodium channels and inhibit HDAC activity [51]. Our study found that VPA promoted Purkinje cell survival by restoring microtubule organization and mitochondrial function (Fig. S15). Purkinje cell degeneration and neuroinflammation are typical symptoms of cerebellar ataxia, and VPA improved symptoms that corresponded to the major domains of ataxia, including motor function, Purkinje cell degeneration and neuroinflammation, in our murine model. Therefore, our data suggest that VPA would be an effective treatment for SCAR20 and other types of cerebellar ataxia. However, the therapeutic potential of VPA for the treatment of cerebellar ataxia warrants rigorous evaluation in future clinical studies in SCAR20 patients.

In summary, our elucidation of SNX14 in the regulation of mitochondrial transport in axons provides new mechanistic insights into the pathogenesis of cerebellar ataxia. Importantly, we propose the chronic administration of the antiepileptic drug VPA as a potential treatment for patients with SNX14 deficiency.

## METHODS

The detailed methods and materials are available as supplementary data.

## Supplementary Material

nwab024_Supplemental_Files
