# Supplementary material for: SNX14 deficiency-induced defective axonal mitochondrial transport in Purkinje cells underlies cerebellar ataxia and can be reversed by valproate
Source: Natl Sci Rev. 2021 Feb 10;8(7):nwab024. doi: 10.1093/nsr/nwab024 (PMC8310771; doi:10.1093/nsr/nwab024)
Supplement: nwab024_Supplemental_Files [file nwab024_supplemental_files.zip › Supplemental data revised 20210118.pdf]

Supplemental information

SNX14 deficiency-induced defective axonal mitochondrial transport in Purkinje cells underlies cerebellar ataxia and can be reversed by valproate

Hongfeng Zhang[1,†], Yujuan Hong[1,†], Weijie Yang[1], Ruimin Wang[1], Ting Yao[1], Jian Wang[1], Ke Liu[2], Huilong Yuan[1], Chaoqun Xu[2], Yuanyuan Zhou[1], Guanxian Li[1], Lishan Zhang[1], Hong Luo[1], Xian Zhang[1], Dan Du[3], Hao Sun[1], Qiuyang Zheng[1], Yun-Wu Zhang[1], Yingjun Zhao[1], Ying Zhou[2], Huaxi Xu[1] and Xin Wang[1,*]

[1]State Key Laboratory of Cellular Stress Biology, Fujian Provincial Key Laboratory of Neurodegenerative Disease and Aging Research, Institute of Neuroscience, School of Medicine, Xiamen University, Xiamen, Fujian, 361102, China

[2]National Institute for Data Science in Health and Medicine, School of Medicine, Xiamen University, Xiamen, Fujian, 361102, China

[3]Cancer Research Center, Department of Stomatology, School of medicine, Xiamen University, Xiamen, Fujian, 361102, China

[†] These authors contributed equally to this work

*Correspondence: wangx@xmu.edu.cn

Supplementary Figures

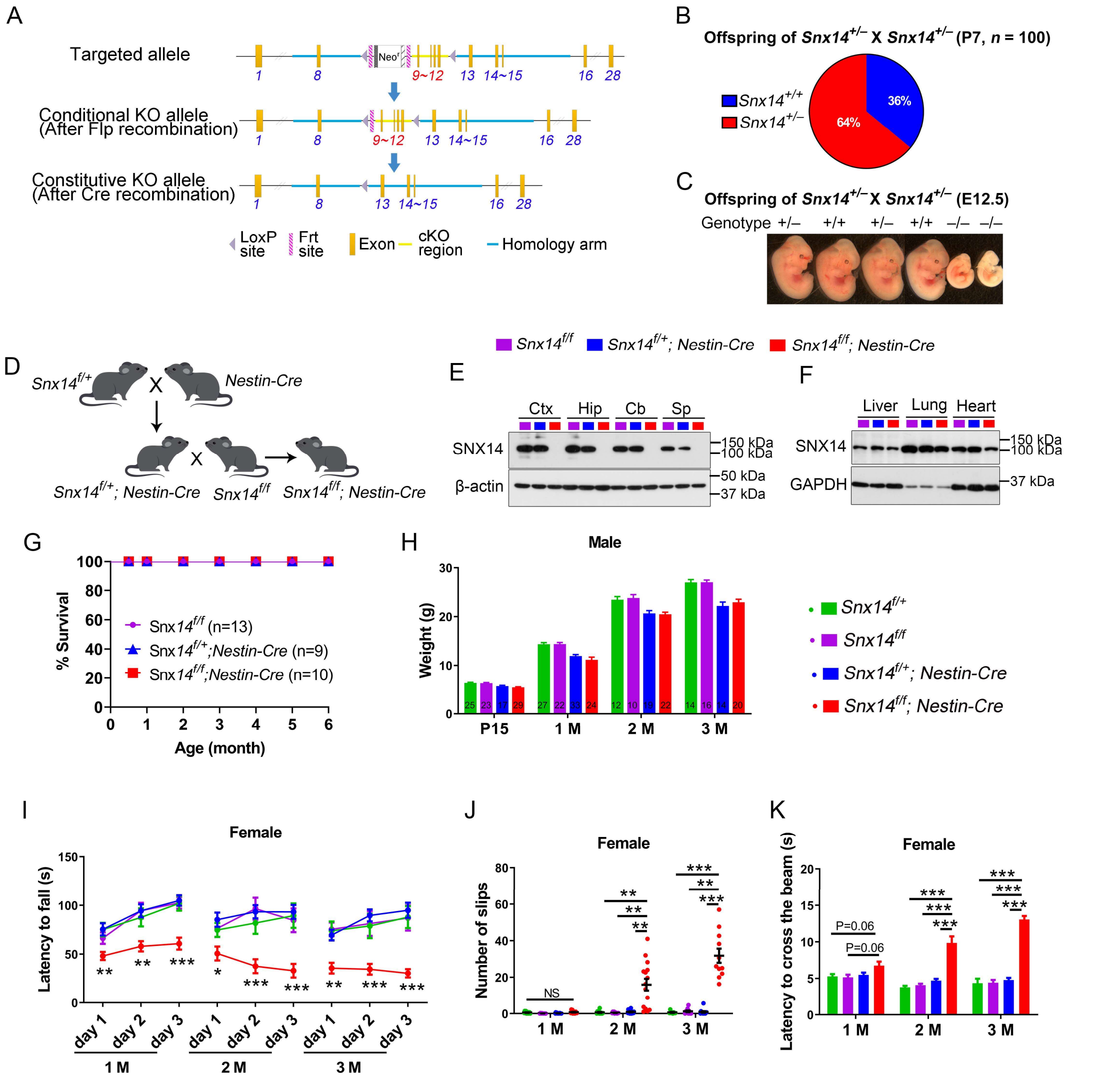

Figure S1. Generation of *Snx14* knockout mice and behavioral analysis. (A) Loxp sites were introduced to flanking exons 9-12 within the *Snx14* gene. (B) The percentage of newborn *Snx14*$^{+/+}$ and *Snx14*$^{+/–}$ pups, 100 animals (postnatal day 7, P7) were scored in total. (C) Representative images showing embryonic day 12.5 (E12.5) *Snx14*$^{+/+}$, *Snx14*$^{+/–}$ and *Snx14*$^{–/–}$ embryos. (D) The breeding strategy of *Snx14*$^{f/f}$*;Nestin-Cre* mice. (E) SNX14 expression in the cortex (Ctx), hippocampus (Hip), cerebellum (Cb) and spinal cord (Sp). (F) SNX14 expression in the liver, lung and heart. (G) Survival analysis of *Snx14*$^{f/f}$, *Snx14*$^{f/+}$*;Nestin-Cre* and *Snx14*$^{f/f}$*;Nestin-Cre* mice. (H) Body weight of *Snx14*$^{f/+}$, *Snx14*$^{f/f}$, *Snx14*$^{f/+}$*;Nestin-Cre* and *Snx14*$^{f/f}$*;Nestin-Cre* mice at different ages. (I) Quantification of the latency to fall in the rotarod test. n = 8~18 mice per genotype per age. (J) Quantification of mouse foot slips and (K) the latency to cross in balance beam tests. n = 8~16 per genotype per age. P15 represents postnatal day 15. 1 M, 2 M, and 3 M represent 1 month, 2 months, and 3 months of age,

respectively. All data represent means ± S.E.M. *P* values were determined using two-sided log-rank (Mantel-Cox) test with multiple comparisons in (G), by repeated-measures ANOVA with Bonferroni's *post hoc* analysis in (I), by one-way ANOVA with Tukey’s *post hoc* analysis in (H) and (J), and by the Kruskal-Wallis test with Dunn’s *post hoc* analysis in (K). NS, not significant; *$P < 0.05$; **$P < 0.01$; ***$P < 0.001$.

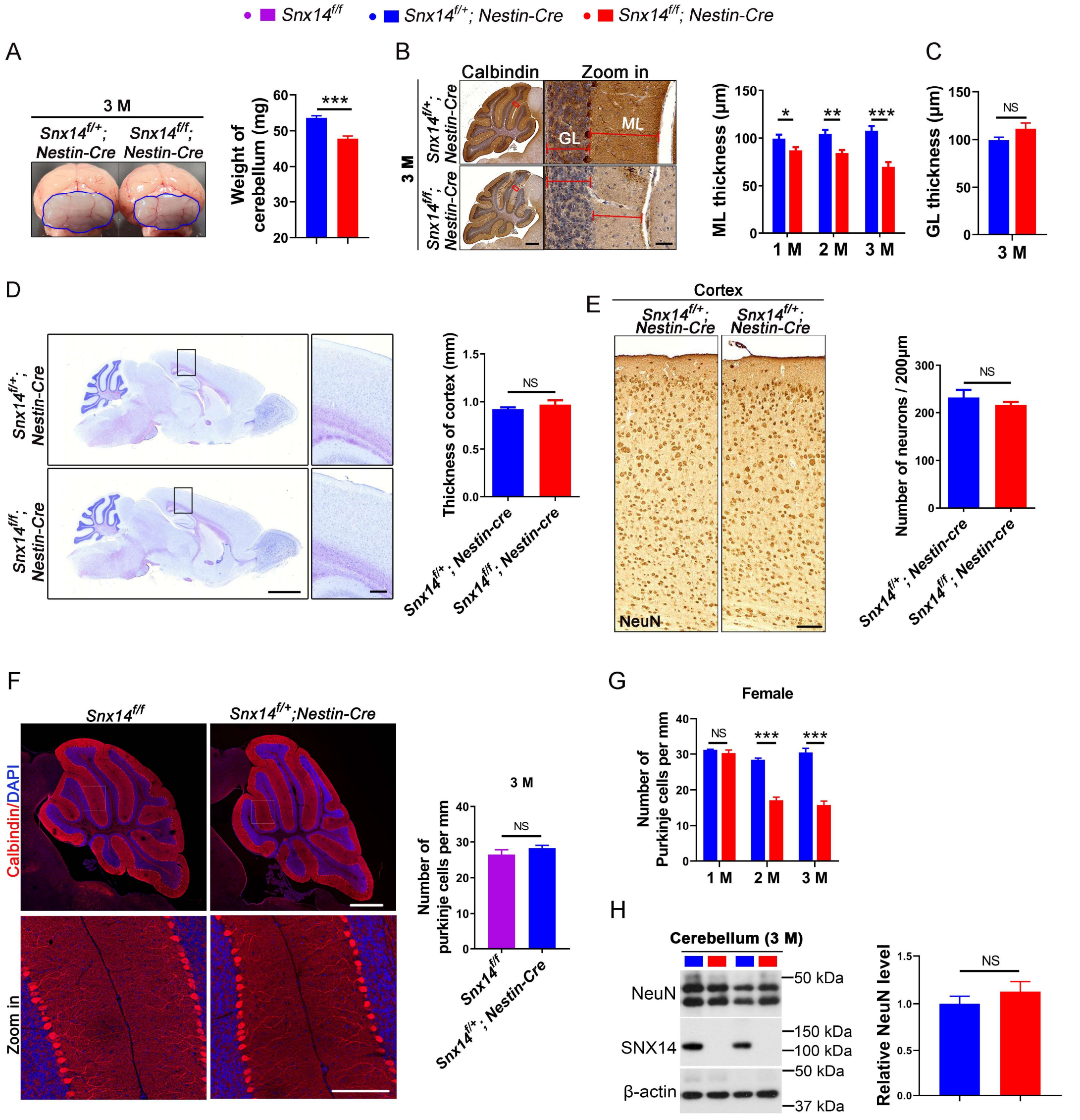

Figure S2. SNX14 deficiency leads to cerebellar degeneration. (A) Representative cerebellar images (blue area) (left) and cerebellar weight (right) in *Snx14$^{f/+}$;Nestin-Cre* and *Snx14$^{f/f}$;Nestin-Cre* mice. *n* = 4~5 mice per genotype per age. (B and C) Immunohistochemical (IHC) staining for calbindin (brown-DAB) counterstained with hematoxylin (blue) and measurement of cerebellar ML (B) and GL thickness (C) in cerebellar sections from *Snx14$^{f/+}$;Nestin-Cre* and *Snx14$^{f/f}$;Nestin-Cre* mice. GL, granular layer; ML, molecular layer. Scale bars = 500 µm (left) and 50 µm (right). *n* = 3 mice. (D) Nissl staining showing whole brains and cerebral cortex of 2-month-old *Snx14$^{f/+}$; Nestin-Cre* and *Snx14$^{f/f}$; Nestin-Cre* mice. Scale bars = 2 mm (left) and 200 µm (right). *n* = 3 mice. (E) IHC labeling and quantification of NeuN^{+} neurons in cerebral cortex of 2-month-old *Snx14$^{f/+}$; Nestin-Cre* and *Snx14$^{f/f}$; Nestin-Cre* mice. Scale bar = 100 µm. *n* = 3 mice. (F) Immunofluorescence staining and quantification of calbindin^{+} Purkinje cells (red) in *Snx14$^{f/f}$* and *Snx14$^{f/+}$;Nestin-Cre* mouse cerebella. Scale bars = 1 mm (top), 200 µm (bottom). *n* = 3 mice per genotype. (G) Quantification of cerebellar Purkinje cells. *n* = 3 mice (7~9 slices in total) per genotype per age. (H) Immunoblot

analysis of NeuN expression in the cerebellum. Signal intensities from immunoblots were calculated and normalized to β-actin. $n = 6$ mice per genotype per age. 1 M, 2 M, and 3 M represent 1 month, 2 months, and 3 months of age, respectively. All data represent means ± S.E.M. P values were determined using Student's t test. NS, not significant; *$P < 0.05$; **$P < 0.01$; ***$P < 0.001$.

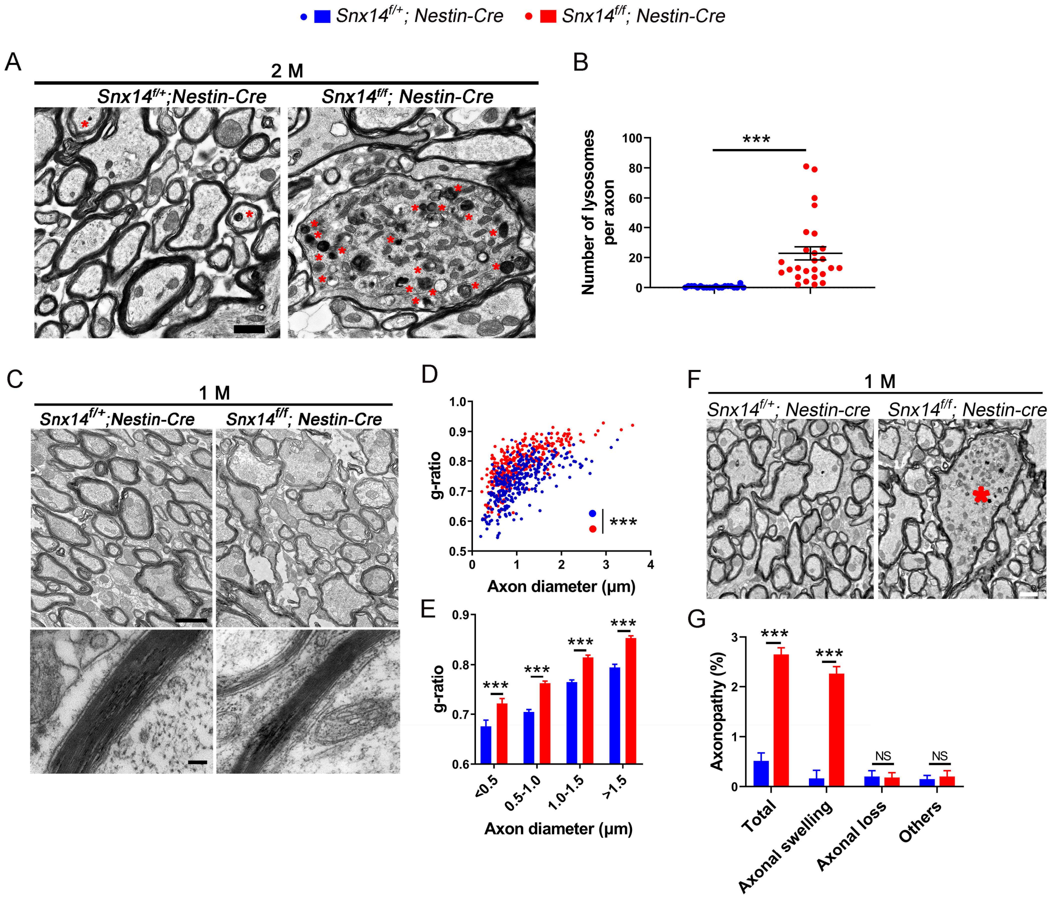

Figure S3. Axonopathy and hypomyelination in the cerebella of *Snx14*-deficient mice. (A) Representative TEM images of Purkinje cell axons. Scale bar = 1 μm. Lysosomes are indicted with red asterisks. (B) Quantification of lysosomes in swollen axons of Purkinje cells from 2-month-old *Snx14$^{f/+}$;Nestin-Cre* (*n* = 4 mice, 20 axons) and *Snx14$^{f/f}$;Nestin-Cre* (*n* = 4 mice, 26 axons) mice. Each plot represents an axon. (C) Representative TEM images of Purkinje cell axons. Scale bars = 1.5 μm (top) and 0.1 μm (bottom). (D) Scatter plot of g-ratio values for Purkinje cell axons in 1-month-old *Snx14$^{f/+}$;Nestin-Cre* (*n* = 3 mice, 322 axons) and *Snx14$^{f/f}$;Nestin-Cre* (*n* = 3 mice, 321 axons) mice. (E) Frequency distribution of Purkinje cell axons according to diameter. (F) Representative TEM images of Purkinje cell axons. Scale bar = 1 μm. The asterisk indicates a swollen axon. (G) The percentage of axonopathies (axonal swelling, axonal loss and others) of Purkinje cells; 400~600 axons per mouse were counted. *n* = 3 mice per group. 1 M and 2 M represent 1 month and 2 months of age. All data represent means ± S.E.M. *P* values were determined using Student's *t* test. NS, not significant; ****P* < 0.001.

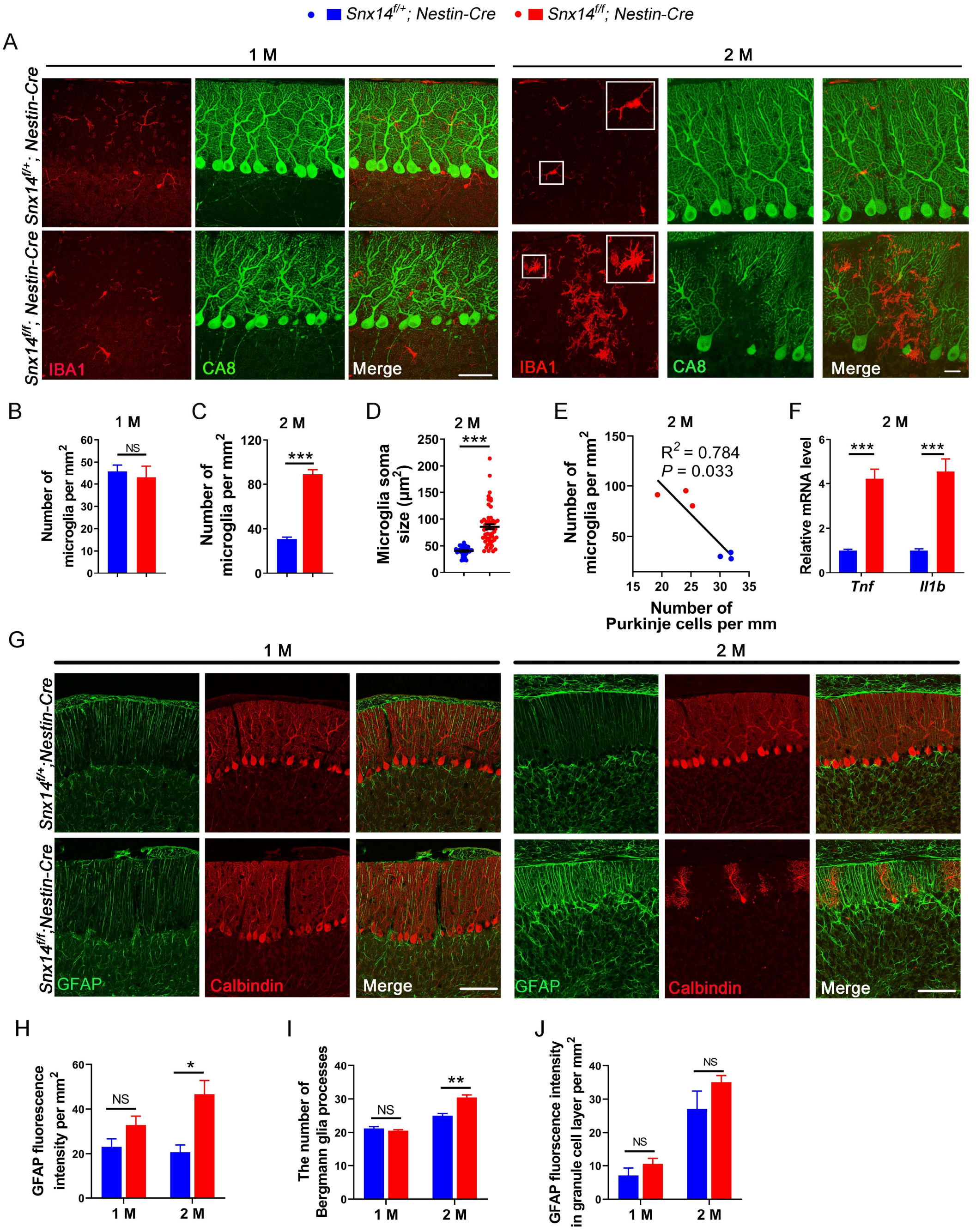

Figure S4. Progressive Purkinje cell loss and glial activation in *Snx14*-deficient mouse cerebellum. (A) Double immunofluorescence staining of Purkinje cells (CA8, green) and microglia (IBA1, red) in 1-month-old and 2-month-old mouse cerebella. Scale bars = 50 μm (left) and 20 μm (right). (B) Quantification of microglia in 1-month-old mouse cerebella. n = 3 mice. (C and D) Quantification of IBA1$^+$ microglial number (C) and soma size (D) in 2-month-old mouse cerebella. n = 3 mice. (E) Correlation between the number of Purkinje cells and microglia in *Snx14$^{f/+}$;Nestin-Cre* (blue dots) and *Snx14$^{f/f}$;Nestin-Cre* (red dots) mouse cerebella. (F) qRT-PCR quantification of *Tnf* and *Il1b* mRNA levels in 2-month-old mouse cerebella. n = 6~7 mice per group. (G)

Double immunofluorescence staining of Purkinje cells (calbindin, red) and Bergmann glia or astrocytes (GFAP, green) in 1-month-old and 2-month-old mouse cerebella. Scale bars = 50 μm. (H-J) Quantification of GFAP fluorescence intensity (H), Bergmann glia processes (I) and GFAP fluorescence intensity in granule cell layer (J) of 1-month-old and 2-month-old mouse cerebella. 1 M and 2 M represent 1 month and 2 months of age. All data represent means ± S.E.M. P values were determined using Student's t test in (B-D), (F), (H-J) and Spearman's rank correlation in (E). NS, not significant; $^{*}P < 0.05$; $^{**}P < 0.01$; $^{***}P < 0.001$.

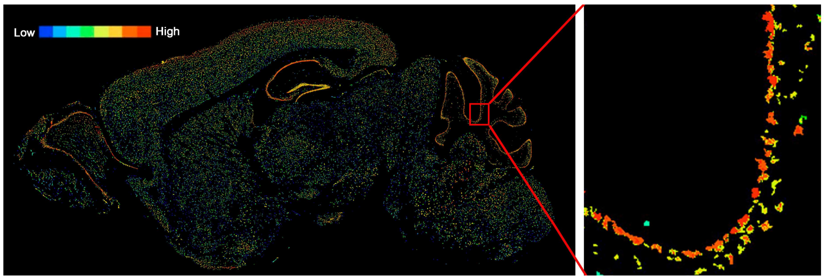

Figure S5. ***Snx14*** **mRNA is highly expressed in the Purkinje cell layer of mouse cerebellum.** Images are reproduced from the Allen Brain Atlas (www.brainatlas.org).

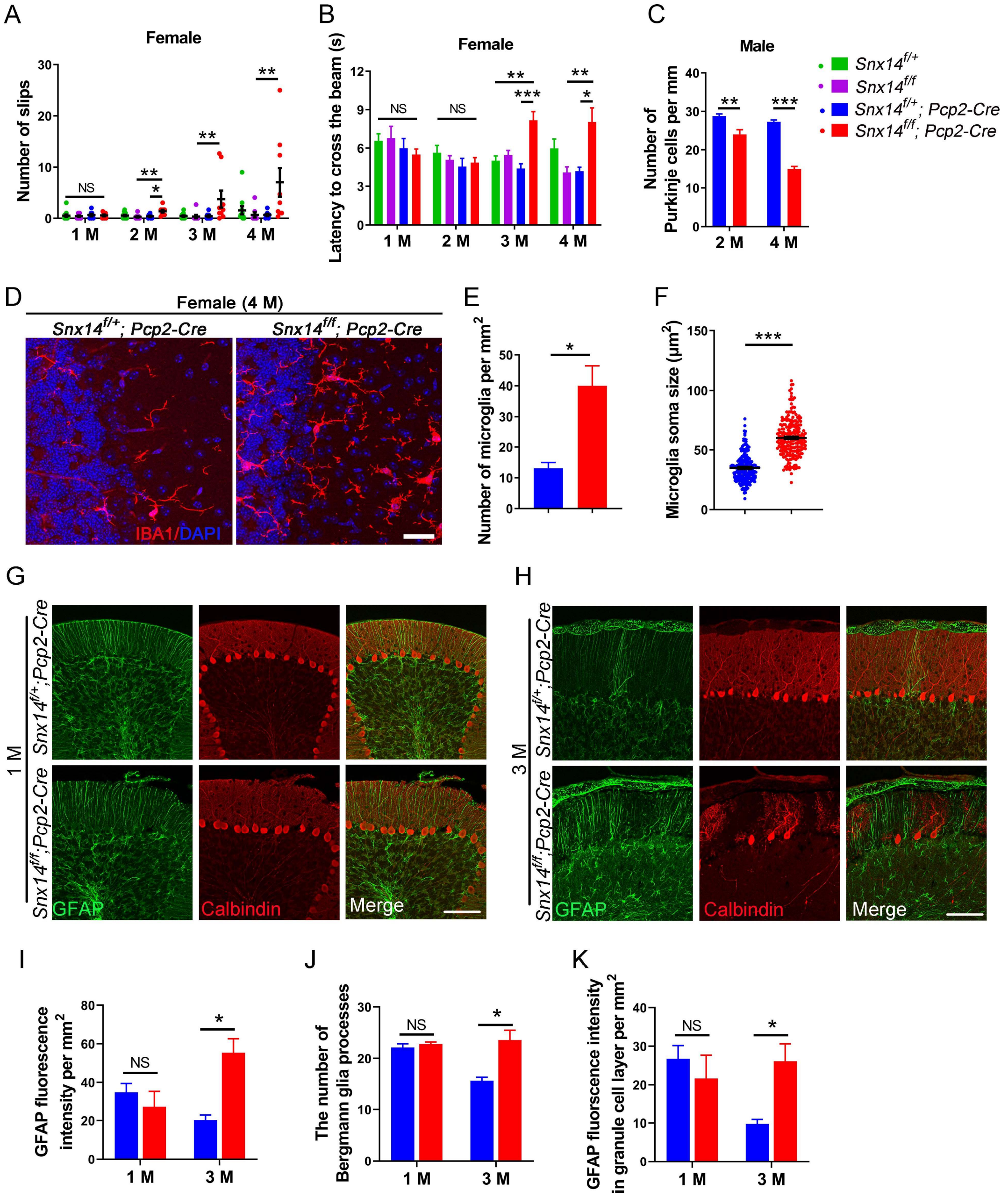

Figure S6. Characterization of motor coordination deficits, Purkinje cell death and glial activation in *Snx14$^{f/f}$;Pcp2-Cre* mice. (A) Quantification of female mouse foot slips and (B) the latency to cross in balance tests. *n* = 8~13 mice per genotype per age. (C) Quantification of cerebellar Purkinje cells in male mice. *n* = 3 mice (total 7~9 slices) per genotype per age. (D) IBA1 immunofluorescence staining of 4-month-old female mouse cerebellar sections. Scale bar = 25 μm. (E) Measurement of the IBA1^{+} cell number. (F) Quantification of microglial cell body size. *n* = 3 mice per group. (G and H) Double immunofluorescence staining of Purkinje cells (calbindin, red) and Bergmann glia or astrocytes (GFAP, green) in 1-month-old (G) and 3-month-old (H) mouse cerebella. Scale bars = 50 μm. (I-K) Quantification of GFAP fluorescence intensity (I), Bergmann glia processes (J) and GFAP fluorescence intensity in granule

cell layer (K) in 1-month-old and 3-month-old mouse cerebella. 1 M, 2M, 3 M and 4 M represent 1 month, 2 months, 3 months and 4 months of age, respectively. All data represent means ± S.E.M. *P* values were determined using the Kruskal-Wallis test with Dunn's *post hoc* analysis in (A), (B) and Student's *t* test in (C), (E), (F) and (I-K). NS, not significant; *$P < 0.05$; **$P < 0.01$; ***$P < 0.001$.

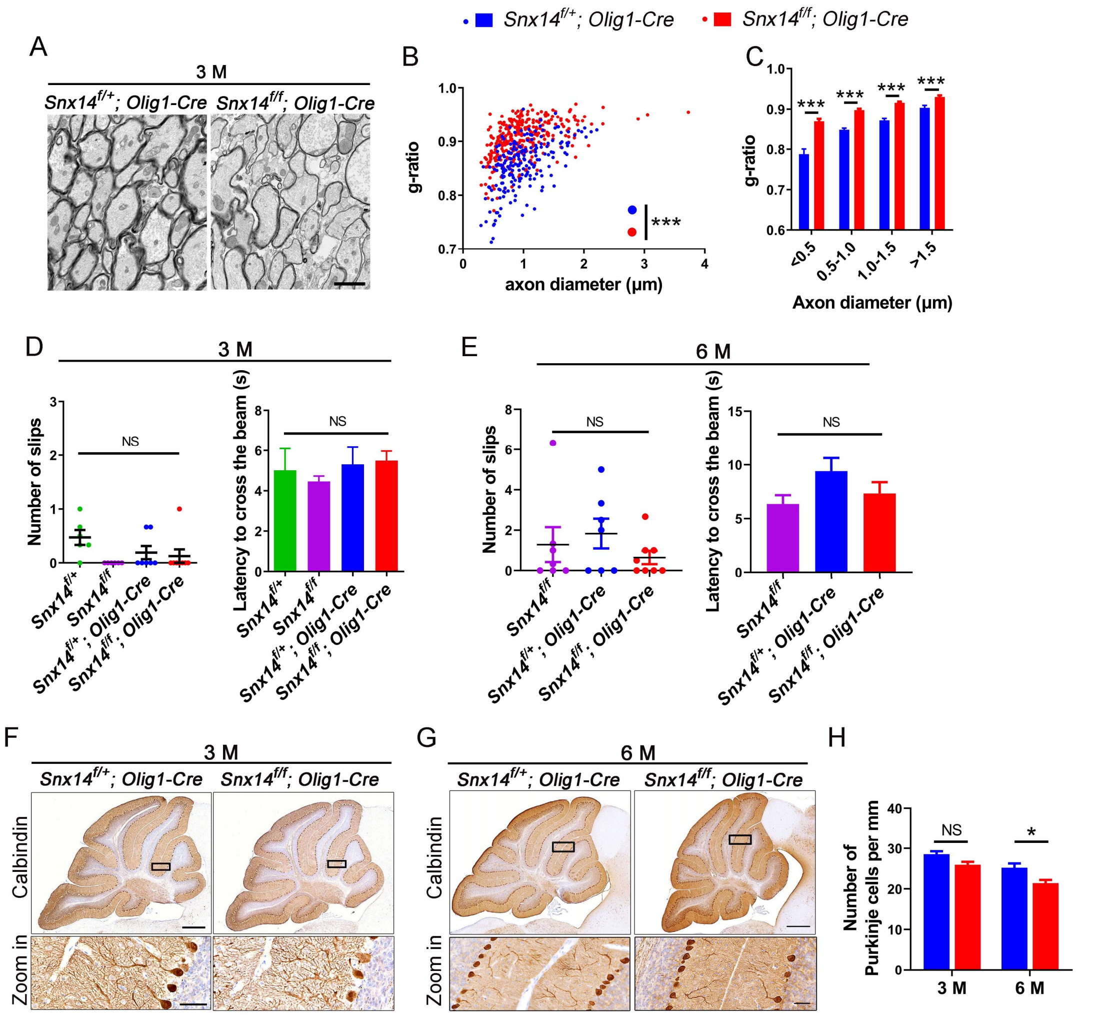

Figure S7. Characterization of neuropathological phenotypes in *Snx14$^{f/+}$;Olig1-Cre* mice. (A) Representative TEM images of Purkinje cell axons from *Snx14$^{f/+}$;Olig1-Cre* and *Snx14$^{f/f}$;Olig1-Cre* mice. Scale bar = 2 μm. (B) Scatter plot of g-ratio values of Purkinje cell axons in *Snx14$^{f/+}$;Olig1-Cre* (202 axons) and *Snx14$^{f/f}$;Olig1-Cre* (303 axons) mice. *n* = 3 mice per genotype. (C) Frequency distribution of Purkinje cell axons according to diameter. (D) Quantification of mouse foot slips (left) and the latency to cross (right) in balance beam tests. *n* = 6~8 per genotype. (E) Quantification of mouse foot slips (left) and the latency to cross (right) in balance beam tests. *n* = 7~8 mice per genotype. (F) Representative images and quantification of calbindin$^+$ Purkinje cells in the cerebella from 3-month-old mice. Scale bars = 500 μm (top) and 50 μm (bottom). *n* = 3 mice per genotype. (G) Representative images and quantification of calbindin$^+$ Purkinje cells in the cerebella from 6-month-old mice. Scale bars = 500 μm (top) and 50 μm (bottom). *n* = 3 mice per genotype. 1 M, 2 M, 3 M, 4 M and 6 M represent 1 month, 2 months, 3 months, 4 months and 6 months of age, respectively. All data represent means ± S.E.M. *P* values were determined using Student's *t* test in (B), (C) and (H), and the Kruskal-Wallis test with Dunn's *post hoc* analysis in (D) and (E). NS, not significant; *P < 0.05; ***P < 0.001.

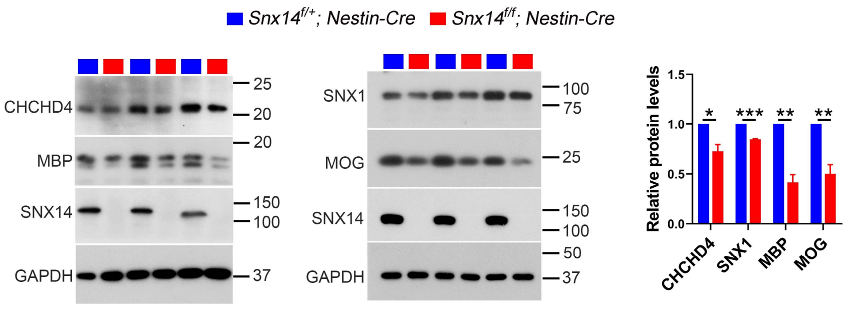

Figure S8. Immunoblot analysis of CHCHD4, MBP, SNX1 and MOG in the cerebella of 1-month-old *Snx14$^{f/+}$;Nestin-Cre* and *Snx14$^{f/f}$;Nestin-Cre* mice. Signal intensities from immunoblots were calculated and normalized to GAPDH. *n* = 3 mice per group. All data represent means ± S.E.M. *P* values were determined using Student's *t* test. **P* < 0.05; ***P* < 0.01; ****P* < 0.001.

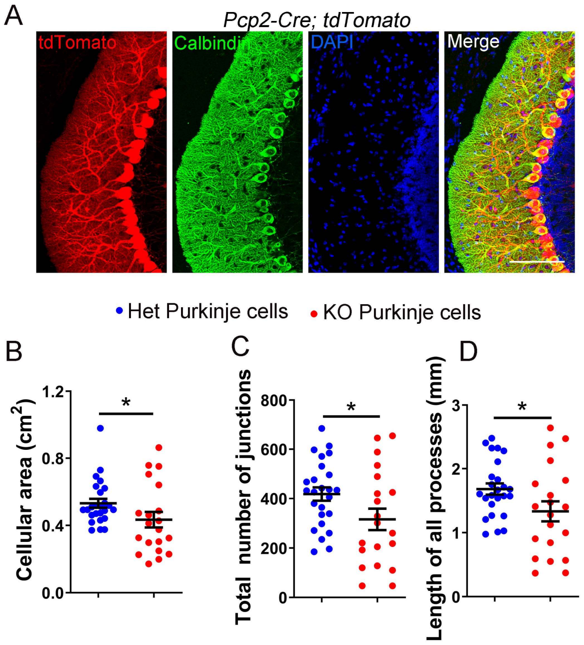

Figure S9. *Snx14*-deficient Purkinje cells feature the abnormal morphology. (A) Immunofluorescence staining of calbindin⁺ Purkinje cells (green) in the *Pcp2-Cre;tdTomato* mouse cerebellum. Scale bar = 100 μm. (B-D) Morphological analysis of Het (n = 25 cells) and KO Purkinje cells (n = 20 cells). The cellular area (B), total number of junctions (C) and the summed length of all processes (D) were quantified. All data represent means ± S.E.M. P values were determined using Student's t test. *P < 0.05.

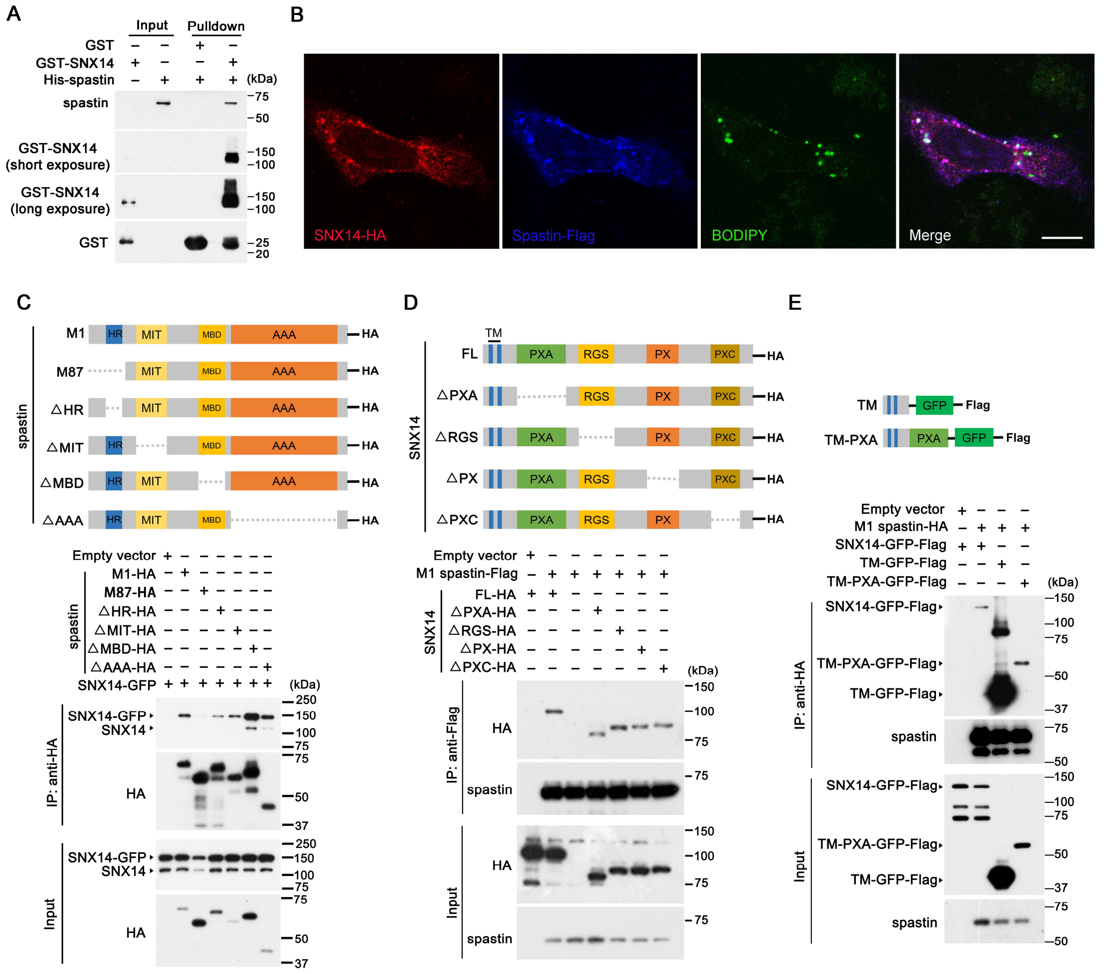

Figure S10. SNX14 interacts with spastin through their N-terminal domains. (A) *In vitro* pull-down assay showing the direct interaction between GST-SNX14 and His tagged-M1 spastin. (B) Confocal microscopy images of HeLa cells overexpressing SNX14-HA (red) and M1 spastin-flag (blue), lipid droplets were stained using BODIPY dye (green). Scale bar = 10 μm. (C) Co-IP between exogenously expressed full-length SNX14-GFP and HA tagged-truncated spastin fragments. IP, immunoprecipitation. HR, hydrophobic region; MIT, microtubule interacting and trafficking domain; MBD, microtubule binding domain; AAA, AAA ATPase domain. (D) Co-IP between exogenously expressed full-length Flag-M1 spastin and HA tagged-truncated SNX14 fragments. FL, full-length, TM, transmembrane; PXA, PX-associated domain; RGS, regulator of G-protein signaling domain; PX, phox homology domain; PXC, sorting nexin, C terminal domain. (E) Co-IP between exogenously expressed M1 spastin-HA and Flag-tagged SNX14-TM-GFP or SNX14-TM-PXA-GFP.

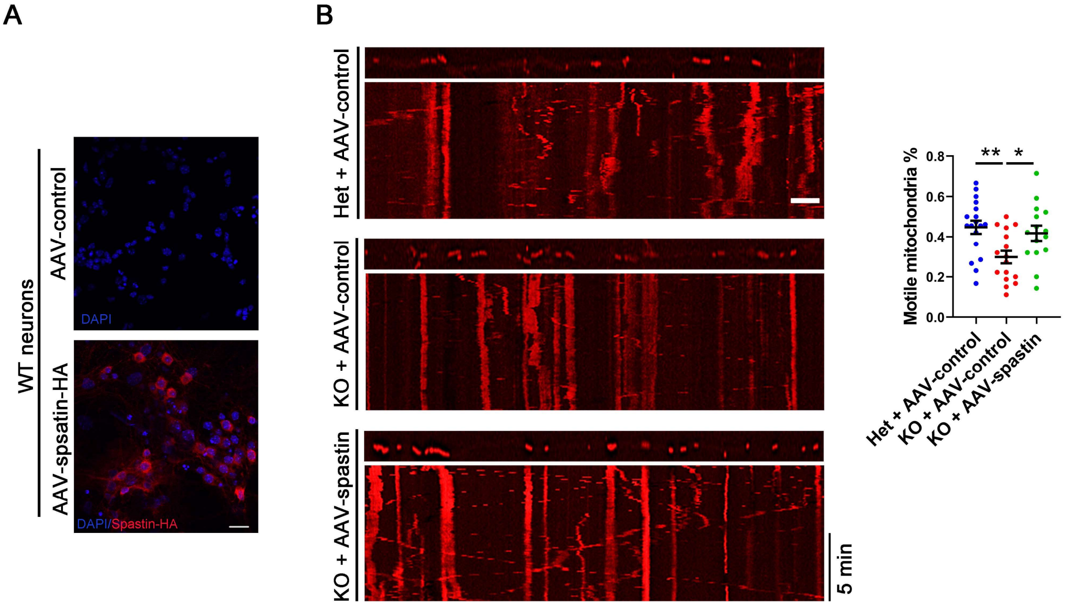

Figure S11. Representative kymographs and quantitative analysis of mitochondrial transport in the axons. (A) Immunofluorescence staining showing spastin-HA expression in WT neurons infected with AAV-control or AAV-spastin-HA. Scale bar = 20 μm. (B) Het and KO neurons were infected with AAV-control or AAV-spastin-HA at DIV3, and mitochondrial motility in the axons was monitored at DIV9 using real-time microscopic imaging. Scale bar = 5 μm. *n* = 15~17 neurons. All data represent means ± S.E.M. *P* values were determined using Student's *t* test. **P* < 0.05; ***P* < 0.01.

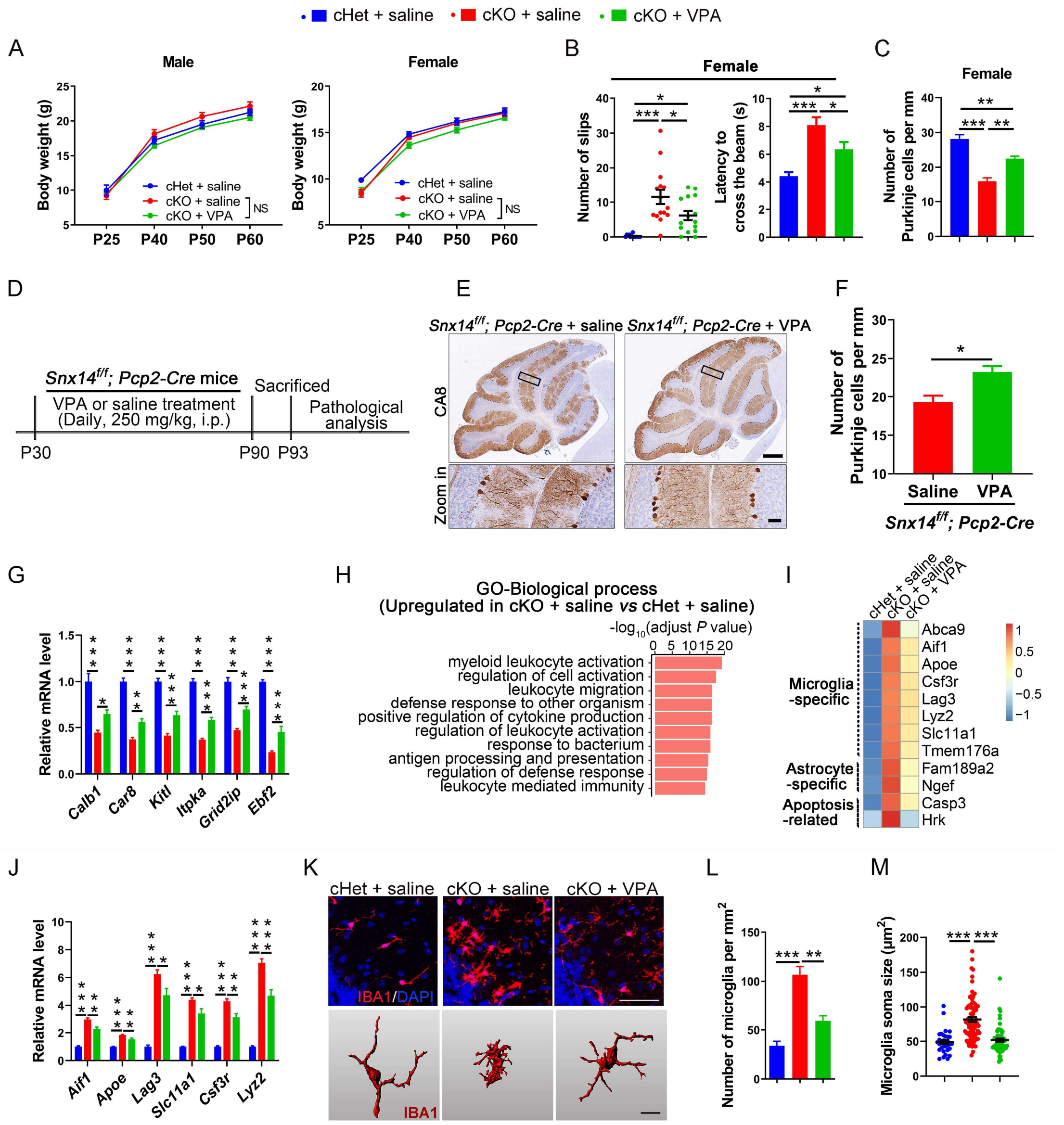

Figure S12. VPA treatment ameliorates cerebellar ataxia, Purkinje cell degeneration and microglial activation. (A) Body weight of male and female cHet + saline, cKO + saline, cKO + VPA mice at different ages. *n* = 8~11 mice per group per age. (B) Quantification of female mouse foot slips (left) and the latency to cross (right) in balance beam tests. *n* = 14~15 mice in each group. (C) Quantification of cerebellar Purkinje cells. *n* = 4~5 mice per group. (D) Schematic timeline of the VPA treatment experiments. *Snx14$^{f/f}$; Pcp2-Cre* mice (P30) were intraperitoneally injected with saline or VPA (250 mg/kg/day) for 2 months, followed by the pathological analysis of mouse cerebellum. (E) Representative IHC images of CA8-positive Purkinje cells in *Snx14$^{f/f}$; Pcp2-Cre* + saline and *Snx14$^{f/f}$; Pcp2-Cre* + VPA mice. Scale bars = 500 μm (top) and 50 μm (bottom). (F) Quantification of Purkinje cells. *n* = 4 mice. (G) qRT-PCR quantification of Purkinje cell-specific gene expression in the mouse cerebellum. *n* = 6~7 mice per group. (H) GO analysis of upregulated genes in the cerebella of cKO + saline mice relative to cHet + saline mice. (I) Heatmap of overlapping genes between

the upregulated genes (cKO + saline v.s. cHet + saline) and the downregulated genes (cKO + VPA v.s. cKO + saline) (fold change > 1.2; adjusted *P* value < 0.1). (J) qRT-PCR quantification of microglia-specific gene expression in the mouse cerebellum. $n = 6$ mice per group. (K) IBA1 immunofluorescence staining and 3-dimensional reconstitution of microglia. Scale bars = 50 µm (top) and 10 µm (bottom). (L) Quantification of the $IBA1^{+}$ cell number. $n = 3$ mice per group. (M) Quantification of the microglial soma size. All data represent means ± S.E.M. *P* values were determined using repeated-measures ANOVA with Bonferroni's *post hoc* analysis in (A), one-way ANOVA with Tukey's *post hoc* analysis in (B), (C), (G), (J), (L) and (M) and Student's *t* test in (F). NS, not significant; *$P < 0.05$; **$P < 0.01$; ***$P < 0.001$.

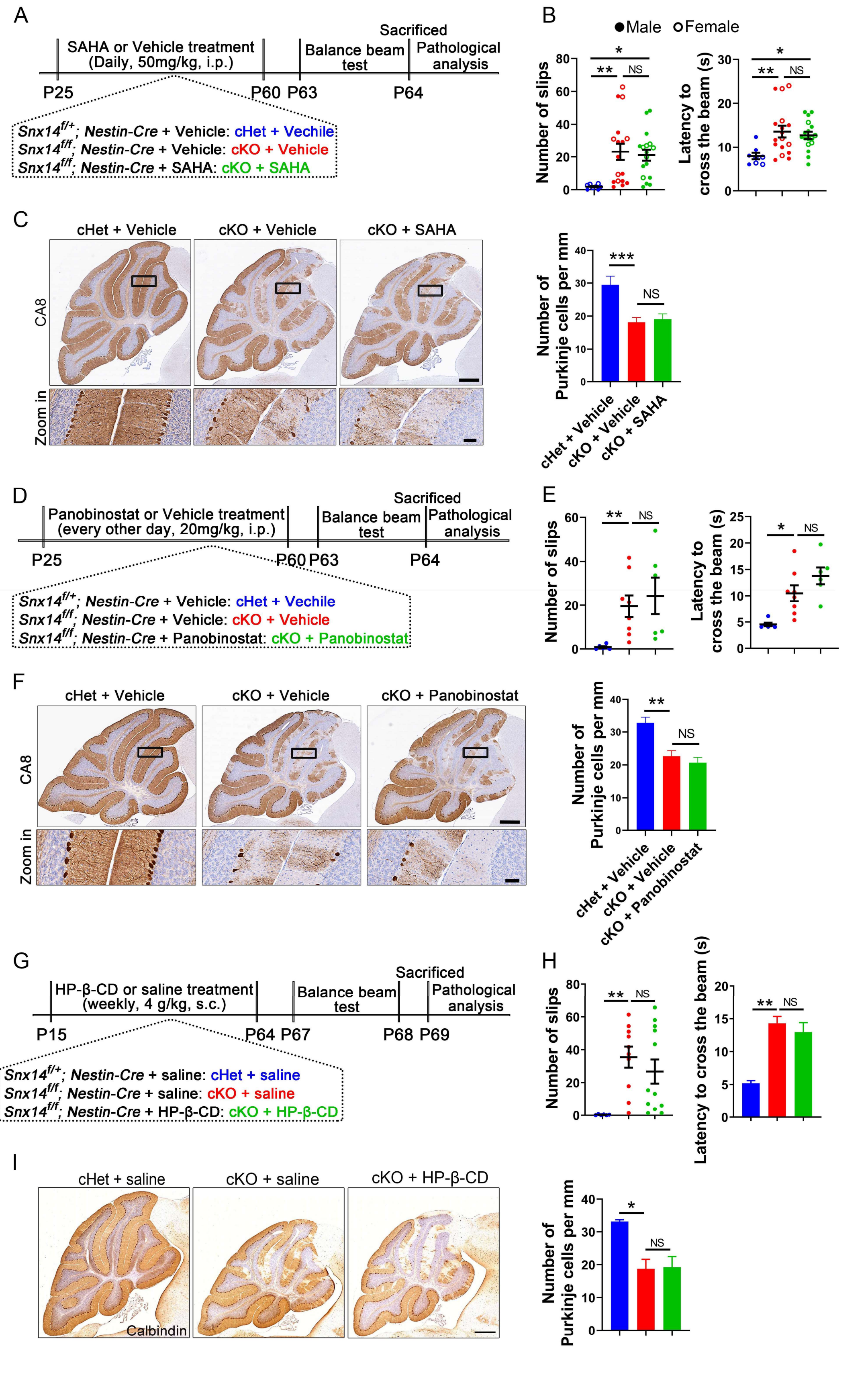

Fig. S13. Administration of HDAC inhibitors or HP-β-CD failed to ameliorate

neurological phenotypes in *Snx14*-deficient mice. (A) Timeline of SAHA treatment experiments. Mice (P25) were intraperitoneally injected with saline or SAHA (50 mg/kg/day) for 5 weeks, followed by behavioral and pathological analysis. (B) Quantification of mouse foot slips (left) and the latency to cross (right) in balance beam tests upon SAHA treatment. n = 8~17 mice per group. (C) Pathological analysis of Purkinje cell survival upon SAHA treatment. Scale bars = 500 μm (top) and 50 μm (bottom). n = 6~10 mice per group. (D) Timeline of Panobinostat administration experiments. Mice (P25) were intraperitoneally injected with saline or Panobinostat (20 mg/kg, every other day) for 5 weeks, followed by behavioral and pathological analysis. (E) Quantification of mouse foot slips (left) and the latency to cross (right) in balance beam tests upon Panobinostat treatment. n = 6~8 mice per group. (F) Pathological analysis of Purkinje cell survival upon Panobinostat treatment. Scale bars = 500 μm (top) and 50 μm (bottom). n = 4~5 mice per group. (G) Timeline of HP-β-CD administration experiments. Mice (P15) were subcutaneously injected with saline or HP-β-CD (4 g/kg/week) for 7 weeks, followed by behavioral and IHC analysis. (H) Quantification of mouse foot slips (left) and the latency to cross (right) in balance beam tests upon HP-β-CD administration. n = 6~12 mice per group. (I) Pathological analysis of the effect of Purkinje cell survival upon HP-β-CD treatment. Scale bar = 500 μm. n = 3 mice per group. All data represent means ± S.E.M. P values were determined using one-way ANOVA with Tukey's *post hoc* test. NS, not significant; *$P < 0.05$; **$P < 0.01$; ***$P < 0.001$.

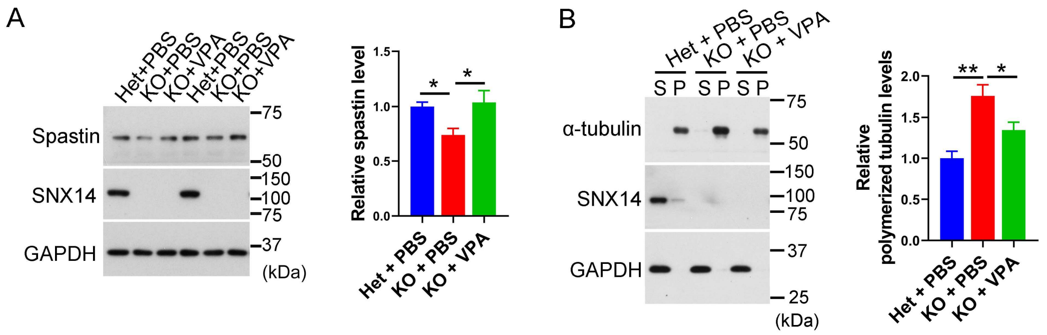

Figure S14. VPA treatment normalized expression of spastin and polymerized α-tubulin in *Snx14* KO neurons. (A) Immunoblot analysis of spastin in cell lysates from *Snx14* Het and KO neurons treated with PBS or VPA. Signal intensities from immunoblots were calculated and normalized to GAPDH. n = 8 per group. (B) Immunoblot analysis of polymerized (P) and soluble (S) α-tubulin extracted from *Snx14* Het and KO neurons treated with PBS or VPA. n = 3~4 per group. All data represent means ± S.E.M. P values were determined using one-way ANOVA with Tukey's *post hoc* test. *P < 0.05; **P < 0.01.

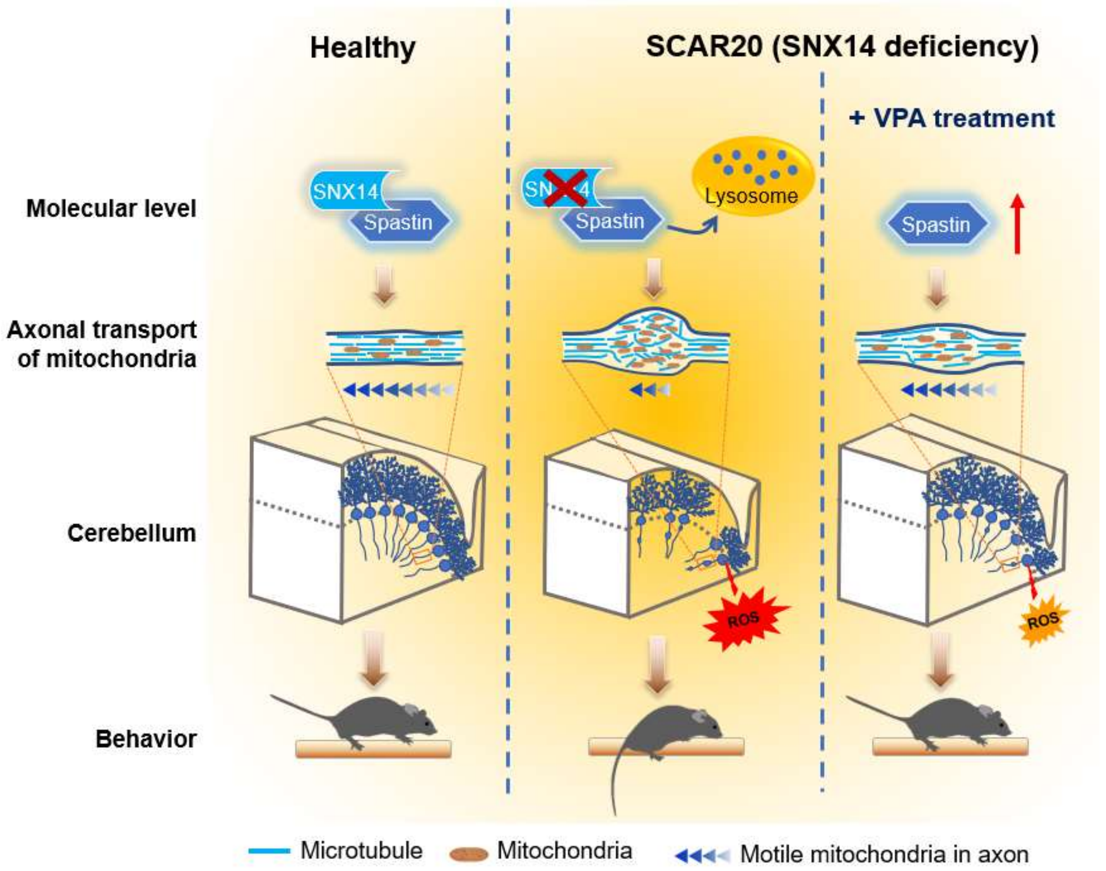

Figure S15. Molecular/cellular mechanism and a potential therapy for SCAR20. SNX14 deficiency impairs microtubule organization and mitochondrial transport in axons via promoting the lysosomal degradation of the microtubule-severing enzyme spastin, thereby compromising axonal integrity and mitochondrial function. Mitochondrial dysfunction further leads to degeneration of the high-energy-demanding Purkinje cells, resulting in the pathogenesis of cerebellar ataxia. The antiepileptic drug valproate ameliorates motor deficits and cerebellar degeneration in *Snx14*-deficient mice through the restoration of spastin-mediated microtubule organization and mitochondrial function in Purkinje cells.

Movie S1. Representative videos of 2.5-month-old female *Snx14*$^{f/f}$, *Snx14*$^{f/+}$*;Nestin-Cre* and *Snx14*$^{f/f}$*;Nestin-Cre* mice in balance beam tests.

Movie S2. Representative videos of axonal mitochondrial transport in neurons from *Snx14*$^{f/+}$*;Nestin-Cre* and *Snx14*$^{f/f}$*;Nestin-Cre* mice. The left side faces toward the soma. Videos were recorded using a super-resolution microscope at 5-s time intervals for 120 frames. Scale bar, 5 μm.

Movie S3. Representative videos of male cHet + saline, cKO + saline and cKO + VPA mice in balance beam tests.

Table S1. siRNAs and primers utilized

siRNAs	Sequence (5'→3')
Control siRNA: sense	UUCUCCGAACGUGUCACGUTT
Control siRNA: antisense	ACGUGACACGUUCGGAGAATT
SNX14 siRNA1: sense	CCAGAUACUGUGAAUCAUUTT
SNX14 siRNA1: antisense	AAUGAUUCACAGUAUCUGGTT
SNX14 siRNA2: sense	GGACAUUGUGAUACAGGAATT
SNX14 siRNA2: antisense	UUCCUGUAUCACAAUGUCCTT
Primers for RT-qPCR	Sequence (5'→3')
Mbp Forward	GACACGGGCATCCTTGACTC
Mbp Reverse	TGAAGAAATGGACTACTGGGTTTT
Cldn11 Forward	CTGGCTGGGGTGCTCCTTA
Cldin11 Reverse	GATGGTGATCTCGCGGTGG
Plp1 Forward	TGGTACAGAAAAGCTAATTGAGACC
Plp1 Reverse	GCCCCATAAAGGAAGAAGAAAGA
Mog Forward	CGGGCTTTAGTTGGGGATG
Mog Reverse	CGGTATTCAGGTGCTTGCTCT
Spast Forward	AGCTGTTCTCAGGCGTTTCAT
Spast Reverse	CTTCCAGAGTATCCATCGGTCAT
Calb1 Forward	TCAGGATGGCAACGGATACA
Calb1 Reverse	TCCAGCAGAAAGAATAAGAGCAA
Car8 Forward	GGGCTTAGTGTTTCCTGATGCT
Car8 Reverse	TGGTCCTCCTGACAAGACTGATT
Grid2ip Forward	GGCTGAAAATGCCTCCCTC
Grid2ip Reverse	AGAATCTGAATCACTGGCGAAA
Kitl Forward	GCCAGCTCCCTTAGGAATGA
Kitl Reverse	GCTCCAAAAGCAAAGCCAAT
Itpka Forward	GAGGACGTGGGGCAGAAAA
Itpka Reverse	CCGCACCAGGCAGTAGTGTT
Ebf2 Forward	GGTGTTTGGGACTATGCTTGTATG
Ebf2 Reverse	GCTCCTTTGCAGAACTGTTTAGATT
Aif Forward	ATGGAGTTTGATCTGAATGGAAATG
Aif Reverse	GCTCTAGGTGGGTCTTGGGAAC
Csf3r Forward	GCGCTGCATCTAAAGCATGT
Csf3r Reverse	GGTGACGGAGAAGGAGTGGTC
Lyz2 Forward	TCTGGGACTCCTCCTGCTTTC
Lyz2 Reverse	TGGTCTCCACGGTTGTAGTTTGTA
Apoe Forward	GCCGTGCTGTTGGTCACAT
Apoe Reverse	TCCCAGGGTTGGTTGCTTT
Lag3 Forward	GGCTTCAATGTCTCCATCACG
Lag3 Reverse	CAGGGCAGCTCCACCCTAG
Slc11a1 Forward	GCTCTGTTCGCAATAGGTTACTTG
Slc11a1 Reverse	GCCTCCCTGCTCCTCGTTAG
Actb Forward	GAGACCTTCAACACCCCAGC
Actb Reverse	ATGTCACGCACGATTTCCC
Primers for genotyping	Sequence (5'→3')
Snx14 KO Forward	*GCCTGTAGCTGACCTGGAACTTAAT*
Snx14 KO Reverse	*GTACCCCAGGCTTCACTGAGTC*
Snx14^{flox} Forward	ATCTCACAGTCCAAATCAGCACCG
Snx14^{flox} Reverse	CTAAGTAATAATGACAATCCAGCTCG
Universal *Cre* Forward	CATATTGGCAGAACGAAAACGC

Universal *Cre* Reverse	CCTGTTTCACTATCCAGGTTACGG
tdTomato Forward	CTGTTCCTGTACGGCATGG
tdTomato Reverse	GGCATTAAAGCAGCGTATCC

Table S2. Top candidates of mass spectrometry analysis of band B

Description	Peptides	Unique peptides	Coverage (%)	Mass (kDa)	Function (from uniport.org)
**RFA1**	133	133	82	70	Binds to single-stranded DNA to form a nucleoprotein complex.
**DDX5**	57	42	61	69	Mediates the alternative regulation of pre-mRNA splicing and transcriptional regulation.
**XRCC6**	44	44	65	69	Plays a role in chromosome translocation.
**LRC47**	32	32	50	63	Functions in RNA binding.
**IF2B3**	63	25	32	58	Binds to the 5'-UTR of the insulin-like growth factor 2 mRNAs
**HNRPQ**	30	5	51	69	Regulates alternative splicing, polyadenylation and other aspects of mRNA metabolism and transport.
**CPSF6**	20	20	33	59	Functions in 3' RNA cleavage and polyadenylation processing.
**GNL3**	34	33	49	61	May be involved in tumorigenesis and stem cell proliferation.

Materials and methods

Experimental mouse models

Snx14 flox (Snx14$^{f/f}$) mice were generated using homologous recombination in embryonic stem cells by Cyagen Biosciences, Inc (Guangzhou, China). To generate *Snx14$^{-/-}$* mice, *Snx14$^{f/f}$* mice were crossed with EIIa-Cre (B6.FVB-Tg(EIIa-cre)C5379Lmgd/J) mice [1]. *Snx14$^{f/f}$* mice were crossed with heterozygous *Cre* lines (*Nestin-Cre* (B6.Cg-Tg(Nes-cre)1Kln/J), *Pcp2-Cre* (Tg(Pcp2-cre)1Amc/J) or *Olig1-Cre* (B6;129S4-*Olig1$^{tm1(cre)Rth}$*/J)) to generate the F1 offspring (heterozygously floxed and *Cre* positive), and the F1 offspring were intercrossed with *Snx14$^{f/f}$* to generate *Snx14* conditional knockout (*Snx14$^{f/f}$;Nestin-Cre, Snx14$^{f/f}$;Pcp2-Cre, Snx14$^{f/f}$;Olig1-Cre*) and

heterozygous (*Snx14$^{f/+}$;Nestin-Cre, Snx14$^{f/+}$;Pcp2-Cre, Snx14$^{f/+}$;Olig1-Cre*) mice. R26-LSL-tdTomato (B6;129S6-*Gt(ROSA)26Sor$^{tm14(CAG-tdTomato)Hze}$/J*) mice were crossed with *Snx14$^{f/f}$;Pcp2-Cre* to generate *Snx14$^{f/f}$;Pcp2-Cre;tdTomato* mice. In most of the experiments, we used heterozygous littermates as a control since they developed and behaved the same as wild-type (WT) mice. Mice were reared in groups of 4~5 in a temperature-and humidity-controlled room with a 12 h light-dark cycle and had *ad libitum* access to food and water. Animals had no experimental exposure before the behavioral tests. Results of animal experiments were collected blindly and recorded according to toe clipping of the experimental mice. Behavioral and morphological analyses were performed in both gender of *Nestin-Cre* and *Pcp2-Cre* mice. In other experiments, male mice were mainly used.

Plasmids, viral vectors and transduction

The cDNA of human M1-spastin and SNX14 with C-terminal HA tags were cloned into pcDNA3.1 vector (Life Technologies) to generate pcDNA3.1-M1-spastin-HA and pcDNA3.1-SNX14-HA vectors. The two plasmids were subsequently used as templates to generate M87 spastin, spastin-ΔHR, ΔMIT, ΔMBD, ΔAAA and SNX14-△PXA, △RGS, △PX, △PXC vectors using the KOD-Plus-Mutagenesis Kit (TOYOBO, SMK-101). The cDNA of human M1-spastin with C-terminal Flag tag was cloned into pcDNA3.1 vector to generate pcDNA3.1-M1-spastin-Flag vector. The cDNA of human SNX14 was cloned into pRLenti-CMV-EGFP vector (Obio Technology, Shanghai, China) to generate pRLenti-CMV-SNX14-EGFP vector.

The mito-7-mCherry DNA sequence was amplified using mKeima-Red-Mito-7 plasmid (Addgene plasmid #56018) as a template and then cloned into pLenti-CMV vector to generate pLenti-CMV-Mito-7-mCherry virus. Human spastin cDNA with a C-terminal HA tag was cloned into a previously reported AAV vector [2]. AAV-spastin-HA vector were packaged into AAV2/9 in HEK293T cells and purified in-house as previously described [3]. Titration of purified viruses were measured using qRT-PCR.

Motor coordination assessment

The rotarod test was performed using a modified protocol [4]. Experimental mice were trained to maintain their balance on a rotarod with a constant speed (4 rpm/min) for 1 min. Mice were subsequently tested on an accelerating rotarod (4~40 rpm over 5 min), and latency to fall off the rod was recorded. The mice were given 3 trials per day for 3 consecutive days.

The balance beam test was performed as previously described [5]. Experimental mice were trained to walk across the raised beam (60 cm in length, 1.2 cm in diameter) 2 times. Each mouse was given three trials. The latency to cross and the number of foot slips were recorded in each trial. A foot slip was counted if either the left or right hindpaw slipped off the beam. The latency to cross and the number of foot slips per mouse were averaged for analysis.

Immunocytochemistry, immunohistochemistry and Nissl staining

Mice were anesthetized with isoflurane, followed by intracardial perfusion. The brains were dissected and postfixed with PFA overnight, followed by dehydration with a graded sucrose solution. For the immunofluorescence staining, the brains were sliced into 15-μm-thick sections using a freezing microtome (Leica CM1950, Germany). After antigen retrieval in 0.01 M citrate buffer (pH 6.0), the brain sections were blocked with 3% normal bovine serum and permeabilized with 0.2% TritonX-100 at room temperature (RT) for 1 h.

The brain sections or coverslips were incubated with primary antibodies for anti-IBA1 (1/250; Wako, No. 019-19741), anti-CA8 (1/50; Santa Cruz, No. sc-166721), anti-GFAP (1/500; Cell Signaling, No. 3670S), anti-HA (1/200, Cell Signaling, No. 2367S) and anti-Flag (1/50, Abclonal, No. AE063) in 1% normal bovine serum and 0.1% TritonX-100 at 4°C overnight. Goat anti-mouse IgG conjugate Alexa Fluor 488 (Thermo Fisher, A-11001, 1/500) and Alexa Fluor 568 (Thermo Fisher, A-11032, 1/500) were used to detect the primary antibody signals. DAPI (Vectorlabs, H-1200) was used to stain the nucleus. Fluorescent images were captured using a laser confocal microscope (Leica SP8). Lipid droplets (LDs) were visualized by staining the cells with BODIPY dye 493/503 (Invitrogen).

For IHC staining, an UltraSensitive SP kit (mouse/rabbit) and a DAB staining kit (MXB biotechnologies) were used according to the manufacturer's protocol. After antigen retrieval, brain sections were incubated with endogenous peroxidase blocking solution for 10 min at RT. Brain sections were blocked with goat serum for 10 min at RT and then incubated with the primary antibodies anti-calbindin (1/250; Cell Signaling, No. 13176S) and anti-NeuN (1/100; Millipore, No. MAB337) at 4°C overnight. Brain sections were further incubated with biotinylated secondary antibodies for 10 min at RT and then incubated with Streptavidin-Peroxidase. To visualize the signals, the sections were incubated with DAB solution for a few minutes, followed by rinsing in 0.01 M PBS to stop the reaction, and then counterstained with hematoxylin, dehydrated in an ethanol series and mounted. For Nissl staining, the brain sections were stained with a Nissl stain (cresyl violet, Beyotime, Nanjing, China) for 20 min at RT. Images were collected using Motic virtual microscopy (Motic VM1, China).

Transmission electron microscopy

Tissue processing was performed as described previously [6]. Briefly, mice were deeply anesthetized and perfused with precooled PBS buffer. Cerebella were dissected and fixed with 2.5% glutaraldehyde in 0.1 M PBS (pH 7.4) at 4 °C for 2.5 h, washed three times with 0.1 M PBS and postfixed in 1% OsO_4 at 4 °C for 2 h. The samples were subsequently dehydrated in an ethanol gradient (30% (v/v) ethanol (15 min), 50% (v/v) ethanol (15 min), 70% (v/v) ethanol (15 min), 90% (v/v) ethanol (15 min), and 100% ethanol (2 × 20 min)) and embedded in Spurr’s resin. Ultrathin (60 nm) sections were then collected on a copper grid, stained with either uranyl acetate or lead citrate and examined using an HT-7800 transmission electron microscope (Hitachi).

Immunoblot analysis

Cultured cells or mouse tissues were lysed in RIPA buffer (containing 150 mM NaCl, 50 mM Tris-HCl, 2 mM EDTA, 1% NP-40, 0.1% SDS, 0.5% sodium deoxycholate) supplemented with protease inhibitors on ice for 30 min, followed by centrifugation at

12,000 rpm for 15 min at 4°C. The supernatant was transferred to another clean tube, and then the protein concentration was measured using a BCA protein assay kit (Thermo Fisher Scientific). After boiling with SDS loading buffer for 5 min, protein lysates containing 10~20 μg protein were resolved by SDS-PAGE and then transferred to PVDF membranes. Immunoblots were probed with the indicated primary antibody: SNX14 (1/1,000; Sigma, No. HPA017639), NeuN (1/1,000; Millipore, No. MAB337), HA (1/1,000; Cell Signaling, No. 2999), spastin (1/1,000; Abcam, No. ab77144), CHCHD4 (1/500; Santa Cruz, No. sc-365137), MBP (1/3,000; Abcam, No. ab40390), SNX1 (1/1,000; Santa Cruz, No. sc-376376), MOG (1/1,000; Santa Cruz, No. sc-166172), α-tubulin (1/30,000; Millipore, No. MABT205), GST (1/1,000, Invitrogen, No. MA4-004), β-actin (1/4,000), or GAPDH (1/4,000) at 4°C overnight, followed by incubation with goat anti-mouse IgG conjugated HRP (Thermo Fisher Scientific, 31430, 1/3,000) or goat anti-rabbit IgG conjugated HRP (Thermo Fisher Scientific, 31460, 1/3,000) secondary antibody at RT for 1 h. Bands were detected by chemiluminescence using ECL solution. The band intensity was quantified using NIH's ImageJ software and normalized to β-actin or GAPDH.

Co-immunoprecipitation (Co-IP)

To determine the critical domains of spasitn that mediate spastin-SNX14 interactions, HEK293T cells were co-transfected with SNX14-GFP and spastin M1-HA, M87-HA, △MIT-HA, △-MBD-HA, △AAA-HA or empty vector for 24 h. To determine the critical domains of SNX14 that mediate the interactions, HEK293T cells were co-transfected with spastin-Flag and SNX14-△PXA-HA, △RGS-HA, △PX-HA or △PXC-HA for 24 h. To determine whether transmembrane domains (TM) of SNX14 mediate the interactions, HEK293T cells were co-transfected with M1 spastin-HA and Flag-tagged SNX14-TM-GFP or SNX14-TM-PXA-GFP 24 h. Then, the cells were lysed in IP buffer (containing 20 mM Tris-HCl, 100 mM NaCl, 1 mM EDTA, 0.5% NP-40) supplemented with protease inhibitors on ice for 30 min, followed by centrifugation at 12,000 rpm for 15 min at 4°C. Lysates were immunoprecipitated using anti-HA magnetic beads (Thermo Fisher Scientific, No. 88837) or anti-Flag antibody (Sigma, No. F1804) plus

protein G magnetic beads (Thermo Fisher Scientific, No. 88848) overnight at 4°C, followed by immunoblot analysis.

In vitro pull-down assay

The cDNA of human M1 spastin and SNX14 were cloned into a pET-His vector and a PGEX-4T-1 bacterial expression vector, respectively. Glutathione S-transferase (GST)-fused SNX14 protein (GST-SNX14) was expressed in *Escherichia coli* BL21 (DE3) strain (C504, Vazyme Biotech) and purified using Glutathione Sepharose 4B beads (GE Healthcare). His tagged-M1 spastin was enriched using nickel-nitrilotriacetic acid-agarose (Qiagen), and washed with Tris buffer containing 20 mM imidazole, and eluted with 200 mM imidazole. For *in vitro* binding assay, the eluted His tagged-M1 spastin protein was incubated with GST or GST-SNX14 proteins which were immobilized on glutathione-conjugated Sepharose beads. The beads were then washed three times and boiled in SDS-PAGE loading buffer for 5 min and followed by immunoblot analysis.

Immunoprecipitation-mass spectrometry

HEK293T cells were transfected with SNX14-HA or empty vector for 24 h. Then, the cells were lysed in IP buffer (containing 20 mM Tris-HCl, 100 mM NaCl, 1 mM EDTA, 0.5% NP-40) supplemented with protease inhibitors on ice for 30 min, followed by centrifugation at 12,000 rpm for 15 min at 4°C. Lysates were immunoprecipitated using anti-HA magnetic beads (Thermo Fisher Scientific, 1/200) overnight at 4°C, followed by immunoblot analysis. After SDS-PAGE, the gels were fixed in 50% methyl alcohol and 10% acetic acid for 15 min at RT. The gels were immersed into 0.02% (w/v) sodium thiosulfate for 2 min, and then incubated with 0.2% (w/v) silver nitrate for 10 min. To visualize the bands, the gels were incubated with the developing solution (containing 6% [w/v] sodium carbonate, 0.05% [v/v] 37% formaldehyde, 0.004% [w/v] sodium thiosulfate) for 5~10 min at RT in the dark. When the bands of interest appeared, the reaction was terminated by discarding the solution and incubated the gel with 6% (w/v) acetic acid for 10 min. The gels were stored in ddH_2O at 4°C until further analysis. The

specific bands were excised and digested, followed by LC-MC/MC analysis using timsTOF Pro (Bruker).

Quantitative reverse transcription polymerase chain reaction (qRT-PCR)

Total RNA was extracted using TRIzol reagent (Invitrogen) according to the manufacturer's instructions. Reverse transcription was performed using Super-Script III Reverse Transcriptase (TOYOBO). The qRT-PCR analysis was carried out using the ROCHE 480 Real-Time LightCycler system and SYBY green reagent (Roche, No. 4913914001); the primer sequence information is included in Table S1. *All* results presented were calculated from CT values derived from the qRT-PCR reactions. All primers for qRT-PCR are listed in Table S1.

High-resolution respirometry

Mitochondrial function in the tissue homogenates from mouse cerebellar tissue was measured using high-resolution respirometry as described previously [7, 8]. Briefly, mice were sacrificed, and the cerebellum was microdissected on ice. Then, the tissue was transferred to ice-cold mitochondrial respiration medium (MiR05) and homogenized in a precooled glass potter. The resulting homogenates containing 2 mg tissue wet weight were suspended in 2.5 ml of ice-cold MiR05, and 2.2 ml of this suspension was used for oxidative phosphorylation (OXPHOS) analysis. Tissue homogenates were transferred into calibrated Oxygraph-2 k (O2k, OROBOROS INSTRUMENTS, Innsbruck, Austria) 2-ml chambers. Oxygen polarography was performed at 37 ± 0.001 °C in O2k-chambers, and oxygen concentration (μM) and oxygen flux per tissue mass (pmol O_2/(s*mg)) were recorded in real-time using DatLab software (OROBOROS INSTRUMENTS, Innsbruck, Austria).

Nonphosphorylating LEAK respiration (CI leak) was induced by the addition of the CI-linked substrates glutamate (10 mM) (Sigma, No. G1626), pyruvate (5 mM) (Sigma, No. P2256), and malate (0.5 mM) (Sigma, No. M1000). Subsequently, the OXPHOS capacity of CI-linked activity (CI_P) was measured after the addition of a saturating concentration of ADP (2.5 mM) (Sigma, No. A5285). OXPHOS capacity with

combined CI and II-linked substrates ($CI\&II_P$) was assessed by the addition of succinate (10 mM). Stepwise titration of FCCP (0.5 μM steps) (MCE, HY-100410) leads to proton leakage through the inner mitochondrial membrane and was used for the measurement of the capacity of the electron transfer system ($CI\&II_E$). Subsequent inhibition of CI by rotenone (0.5 μM) (MCE, HY-B1756) provided a measurement of CII-linked electron transfer system (ETS) capacity (CII_E). To control for other oxygen-consuming processes, CIII was inhibited by antimycin A (Sigma, No. A8674). The resulting residual oxygen consumption (ROX) reflected oxygen consumption from undefined sources and was subtracted from mitochondrial respiratory states. The O_2 flux obtained in each step of the protocol was normalized by the protein concentration of the sample.

Tandem mass tagging (TMT)-based quantitative proteomics

One-month-old male *Snx14$^{f/+}$;Nestin-Cre* and *Snx14$^{f/f}$;Nestin-Cre* mouse cerebella were lysed by sonication in urea buffer. After trypsin digestion, the peptides were labeled using TMT 6-plex reagent (90061, Thermo Fisher Scientific) and subsequently subjected to NSI source, followed by tandem mass spectrometry (MS/MS) in Q Exactive^{TM} Plus (Thermo Fisher Scientific). The resulting spectra were searched against the Proteome Mouse database concatenated with the reverse decoy database. Differential expression was defined by a fold change > 1.2 and *P* value < 0.05.

RNA-seq analysis

Total RNA was extracted from the cerebella by TRIzol reagent (Invitrogen). RNA integrity was measured using the Agilent RNA 6000 Nano Kit (5067-1511, Agilent Technologies, USA). Sequencing libraries were generated using the NEBNext® Ultra^{TM} RNA Library Prep Kit for Illumina® (NEB, USA). Sequencing was carried out using the Illumina Hiseq X TEN platform. Differential expression analysis was performed on the count data using the DESeq2 R package (version 1.16.1). The resulting *P* values were adjusted using the Benjamini-Hochberg procedure for controlling the false discovery rate (FDR). Differentially expressed genes (DEGs) were

defined according to an adjusted *P* value < 0.1 and fold change > 1.2. Gene Ontology (GO) enrichment analysis of DEGs was performed in the cluster Profiler R package, in which the gene length bias was corrected. GO terms with a corrected *P* value < 0.05 were defined as significantly enriched by DEGs.

Purkinje cell sorting and culture

Purkinje cell sorting and culture were performed using a modified protocol [9]. Cerebellar tissues from P6 *Snx14*$^{f/+}$*;Pcp2-Cre;tdTomato* and *Snx14*$^{f/f}$*;Pcp2-Cre;tdTomato* mice were removed and chopped into small pieces. The tissues were then incubated with 0.025% trypsin and 10 kU/ml DNase (37 °C) for 20 min, and the reaction was stopped by the addition of one volume of dissociation solution containing 10% fetal bovine serum. After the samples were centrifuged at 800 g for 5 min, the cell pellet was reserved and resuspended in Hanks balanced salt solution (HBSS) buffer containing 1% fetal bovine serum followed by passage through a 5 ml tube with a cell-strainer cap. The cell suspension was filtered through a 70 μm nylon mesh, and cell sorting was performed with the MoFlo Astrios EQS (Beckman, USA). Cells were passed through a 70 μm nozzle. The sorting decision was based on the forward scatter characteristic (FSC), side scatter characteristic (SCC) and tdTomato fluorescence measurements. The isolated tdTomato^{+} Purkinje cells were plated on glass bottom dishes coated with poly-D-lysine and grown in culture medium (DMEM/F12 containing 4% B27, 1% penicillin/streptomycin, 1% FBS and 0.01% transferin). Half of the old medium was replaced with fresh, warm culture medium every 3 days.

Reactive oxygen species (ROS) assay

The fluorescent probe 2,7-dichlorofluorescein diacetate (DCFH-DA) (Beyotime, Nanjing, China) was used to detect the level of cytosolic ROS in Purkinje cells as previously described [10]. In brief, cells were loaded with 10 μM DCFH-DA for 20 min at 37 °C. After the medium was removed, the cells were carefully washed in DMEM/F12 three times. DCF fluorescence (green) had an excitation of approximately 488 nm, and images were obtained with a laser confocal microscope (Leica SP8). The

fluorescence intensity was quantified using NIH ImageJ.

Mitochondrial motility study

Live cell imaging was performed as described previously [11, 12]. Briefly, cortical neurons from *Snx14$^{f/+}$;Nestin-Cre* and *Snx14$^{f/f}$;Nestin-Cre* mice were isolated and cultured. At 3 days *in vitro* (DIV3), cortical neurons were infected with mitochondria-tagged mCherry lentivirus (pLenti-CMV-Mito-7-mCherry). We subsequently performed time-lapse imaging of cortical neurons at DIV9 for a total of 120 frames with 5 s intervals using a super-resolution microscope (GE DeltaVision OMX). Axons were straightened by ImageJ with the "Straighten" tool, and kymographs were generated with the "Kymograph" tool. The height of the kymographs represents the recording time, and the width of the kymographs represents the length (μm) of the axon imaged. A mitochondrion was considered stationary if its initial and final position was the same. Retrograde transportation was defined by movements toward the soma and displacement > 5 μm, and opposite-direction movements were defined as anterograde transportation.

RNA interference

Small interfering RNAs (siRNAs) were used for SNX14 knockdown. In brief, siRNAs and RNAiMAX Reagent (Thermo Fisher Scientific, No. 13778150) were diluted in Opti-MEM medium. Then, the diluted siRNAs were mixed with the diluted RNAi MAX, followed by a 5 min incubation at RT. Finally, the mix was added to HEK293T cells and incubated for 2 days at 37°C. The SNX14 siRNA and control siRNA sequences are listed in Supplemental Table 1.

Pharmacological treatment of proteasomal and lysosomal inhibitors

HEK293T cells transfected with SNX14 siRNA or control siRNA were incubated for 10 h with 0.1% dimethylsulfoxide (DMSO), proteasomal inhibitor MG132 (10 μM) (MCE, HY-13259) or lysosomal inhibitor leupeptin (100 μg/ml) (MCE, HY-18234A). After treatment, cells were lysed and subjected to western blot analysis.

Purification of polymerized α-tubulin

Monomeric and polymeric tubulin were differentially extracted from cortical neurons using a modified method as previously described [13]. In brief, SNX14 Het and KO cortical neurons were lysed in microtubule (MT)-stabilizing buffer (0.1M PIPES, PH 6.9, 5M $MgCl_2$, 2mM EGTA, 2M Glycerol, 4μM Taxol, and protease inhibitors). Lysates were then centrifuged at 13,000 g for 10 min at room temperature. Supernatants containing solubilized tubulin were transferred to new tubes and 5 X loading buffer was added, the remaining pellets containing sedimented polymerized tubulin were resuspended in 2×loading buffer. Samples were denatured at 95˚C for 5 min and subjected to immunoblot analysis.

Statistical analysis

All statistical analyses were performed using GraphPad Prism 8 software. Values are presented as the means ± SEM. The sample size, the statistical approaches and significance are annotated in the Figures or Figure legends. According to assumptions of normality and equal variance, unpaired student's *t* tests (two-tailed) or Mann-Whitney tests were used for comparisons between two groups, and one-way ANOVA with Tukey *post hoc* analysis or Kruskal Wallis test with Dunn's *post hoc* analysis were used for comparisons of more than two groups. The *P* values in the rotarod tests were determined using repeated-measures ANOVA with Bonferroni's post hoc analysis. Spearman rank correlation was used to analyze the non-normally distributed data.

Study approval

All experimental procedures and animal housing in this study were designed and conducted in accordance with and with the approval of the Institutional Animal Care and Use Committee of Xiamen University.

Reference

1. Lakso M, Pichel JG, Gorman JR, et al. Efficient in vivo manipulation of mouse genomic sequences at the zygote stage. *Proc Natl Acad Sci U S A* 1996;**93**(12):5860-5. doi: 10.1073/pnas.93.12.5860.
2. Zhao YJ, Tseng IC, Heyser CJ, et al. Appoptosin-Mediated Caspase Cleavage of Tau Contributes to Progressive Supranuclear Palsy Pathogenesis. *Neuron* 2015;**87**(5):963-975. doi: 10.1016/j.neuron.2015.08.020.
3. Challis RC, Kumar SR, Chan KY, et al. Systemic AAV vectors for widespread and targeted gene delivery in rodents. *Nature Protocols* 2019;**14**(2):379-414. doi: 10.1038/s41596-018-0097-3.
4. Watase K, Gatchel JR, Sun Y, et al. Lithium therapy improves neurological function and hippocampal dendritic arborization in a spinocerebellar ataxia type 1 mouse model. *PLoS Med* 2007;**4**(5):e182. doi: 10.1371/journal.pmed.0040182.
5. Luong TN, Carlisle HJ, Southwell A, et al. Assessment of motor balance and coordination in mice using the balance beam. *J Vis Exp* 2011(49). doi: 10.3791/2376.
6. Zeng F, Ma X, Zhu L, et al. The deubiquitinase USP6 affects memory and synaptic plasticity through modulating NMDA receptor stability. *PLoS Biol* 2019;**17**(12):e3000525. doi: 10.1371/journal.pbio.3000525.
7. Burtscher J, Bean C, Zangrandi L, et al. Proenkephalin Derived Peptides Are Involved in the Modulation of Mitochondrial Respiratory Control During Epileptogenesis. *Front Mol Neurosci* 2018;**11**:351. doi: 10.3389/fnmol.2018.00351.
8. Burtscher J, Zangrandi L, Schwarzer C, et al. Differences in mitochondrial function in homogenated samples from healthy and epileptic specific brain tissues revealed by high-resolution respirometry. *Mitochondrion* 2015;**25**:104-12. doi: 10.1016/j.mito.2015.10.007.
9. Tomomura M, Rice DS, Morgan JI, et al. Purification of Purkinje cells by fluorescence-activated cell sorting from transgenic mice that express green fluorescent protein. *European Journal of Neuroscience* 2001;**14**(1):57-63. doi: DOI 10.1046/j.0953-816x.2001.01624.x.
10. Esteban MA, Wang T, Qin BM, et al. Vitamin C Enhances the Generation of Mouse and Human Induced Pluripotent Stem Cells. *Cell Stem Cell* 2010;**6**(1):71-79. doi: 10.1016/j.stem.2009.12.001.
11. Zheng YR, Zhang XN, Wu XL, et al. Somatic autophagy of axonal mitochondria in ischemic neurons. *Journal of Cell Biology* 2019;**218**(6):1891-1907. doi: 10.1083/jcb.201804101.
12. Kang JS, Tian JH, Pan PY, et al. Docking of axonal mitochondria by syntaphilin controls their mobility and affects short-term facilitation. *Cell* 2008;**132**(1):137-48. doi: 10.1016/j.cell.2007.11.024.
13. Ng DC, Lin BH, Lim CP, et al. Stat3 regulates microtubules by antagonizing the depolymerization activity of stathmin. *J Cell Biol* 2006;**172**(2):245-57. doi: 10.1083/jcb.200503021.
